# Nanoscale Spatial Organization of ARC High‐ and Low‐Order Assemblies at Excitatory Synapses

**DOI:** 10.1002/advs.202520740

**Published:** 2026-04-07

**Authors:** Martina Damenti, Giovanna Coceano, Mariline Mendes Silva, Jonatan Alvelid, Chiara Sgattoni, Andrea Volpato, Lea Rems, Luciano A. Masullo, Eduard M. Unterauer, Rafal Kowalewski, Lucie Delemotte, Erdinc Sezgin, Ralf Jungmann, Ilaria Testa

**Affiliations:** ^1^ Department of Applied Physics and Science for Life Laboratory KTH Royal Institute of Technology Stockholm Sweden; ^2^ Faculty of Electrical Engineering University of Ljubljana Ljubljana Slovenia; ^3^ Max Planck Institute of Biochemistry Planegg Germany; ^4^ Faculty of Physics and Center for NanoScience Ludwig Maximilian University München Germany; ^5^ Department of Women's and Children's Health Karolinska Institutet Stockholm Sweden

**Keywords:** AMPA receptors, ARC (Activity‐Regulated Cytoskeleton‐associated protein), endocytosis, membrane interaction, oligomerization, Super Resolution Microscopy

## Abstract

The Activity‐Regulated Cytoskeleton‐Associated protein (ARC) plays a pivotal role in mediating synaptic plasticity in neuronal cells. In vitro studies suggest that ARC can form both high‐ and low‐order oligomers. Despite their potentially important functions, direct nanoscale observation of ARC assemblies in cells has been lacking due to its tightly regulated spatiotemporal expression, the small size of the structures, the absence of suitable labelling strategies, and background signals from freely diffusing cytosolic proteins. Here, we combine super‐resolution microscopy and time‐resolved fluorescence anisotropy measurements with precisely designed ARC‐tagging strategies to reveal the nanoscale spatial organization of ARC in neuronal environments, with a particular focus on excitatory synapses. We identify low‐order assemblies of ARC at synapses, that colocalize with surface AMPA receptors (AMPARs), semi‐circular organizations of ARC at the endocytic zone, consistent with a role in mediating AMPAR endocytosis, and particle‐like assemblies sized 60–80 nm predominantly localized in dendritic spines. Finally, using a combination of experiments and simulations, we show that ARC can directly induce membrane bending and lipid bilayer tubulation in the absence of other protein partners.

## Graphical abstract

ARC (Activity‐Regulated Cytoskeleton‐Associated protein) mediates synaptic plasticity by forming nanoscale assemblies in neurons. Using super‐resolution microscopy and time‐resolved anisotropy with targeted tagging, the study reveals low‐order ARC assemblies at synapses colocalizing with AMPARs, semi‐circular structures at endocytic zones, and 60–80 nm particles in dendritic spines. ARC can also directly bend membranes, suggesting roles in AMPAR trafficking and endocytosis.

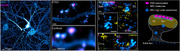

## 1 Introduction

Arc is an immediate early gene transcribed in response to neuronal activity [1, 2, 3, 4, 5, 6]. The protein ARC has been implicated in several functions, including the endocytosis of ionotropic AMPA receptors (AMPARs), thereby mediating homeostatic synaptic scaling and long‐term depression. Consistent with this, ARC interacts with multiple endocytic proteins, such as Endophilin‐3, Dynamin‐2 [7], AP‐2 [8], as well as with the GluA auxiliary subunit TARPγ2 [7, 9, 10, 11]. In addition, ARC influences the transcription of the GluA1 subunit of AMPARs within the nucleus [12] and has been suggested to stabilize the actin cytoskeleton of dendritic spines [13, 14], implying a potential role in long‐term potentiation, although this remains debated [15]. These seemingly opposing roles have long puzzled neuroscientists.

Phylogenomic analyses have revealed that the tetrapod Arc gene shares homology with the Ty3‐Gypsy retrotransposon family. In vitro experiments have demonstrated that ARC can assemble into structures ranging from dimers to higher‐order oligomers, including 32‐mers [16, 17, 18, 19, 20], and virus‐like capsids composed of approximately 130 units [19, 21, 22, 23, 24] under specific experimental conditions. Moreover, ARC has been detected in exosome‐sized extracellular vesicles, which are released and subsequently taken up by recipient neurons [19, 25]. These findings raise the possibility that distinct ARC oligomeric states may support different functions.

In various heterologous systems, we [26] and Hedde et al. [27] have shown that ARC forms not only monomers and low‐order oligomers but also rigid nanometric particles. In primary cortical neurons and in the rat dentate gyrus [28], ARC was found to form low‐abundance dimers and low‐order oligomers (e.g., trimers, tetramers), which increase upon induction of synaptic plasticity. Furthermore, a phosphomimetic ARC mutant that prevents the formation of 32‐mers in vitro was shown to weaken the magnitude of LTD [20]. However, direct visualization and quantification of different fine spatial organization of ARC in neurons – and its potential functional implications – remains challenging due to the complex spatiotemporal regulation and numerous interaction partners of ARC [29].

Here, we provide direct visualization of the various forms of nanoscale organization of ARC in neuronal cells. These include fast, freely diffusing low‐order assemblies; low‐order assemblies localized to the post‐synaptic density; semi‐circular organizations observed at the endocytic zone; and larger particle‐like assemblies (60–80 nm in diameter) primarily found in dendritic spines, with sizes comparable to viral‐like capsids previously observed in vitro using purified proteins. We also investigate with simulation‐based predictions and experiments in simplified systems the ability of ARC to interact with and bend lipid bilayers independently of other known interaction partners. Finally, by expressing an oligomerization‐incompetent truncated ARC, we resolve a significantly altered spatial organization compared to full‐length ARC: the mutant does not show synaptic assemblies and affects the synaptic surface level of AMPAR.

## 2 Results

### 2.1 ARC is Organized in Nanoclusters in Primary Neuronal Cultures

**FIGURE 1:**
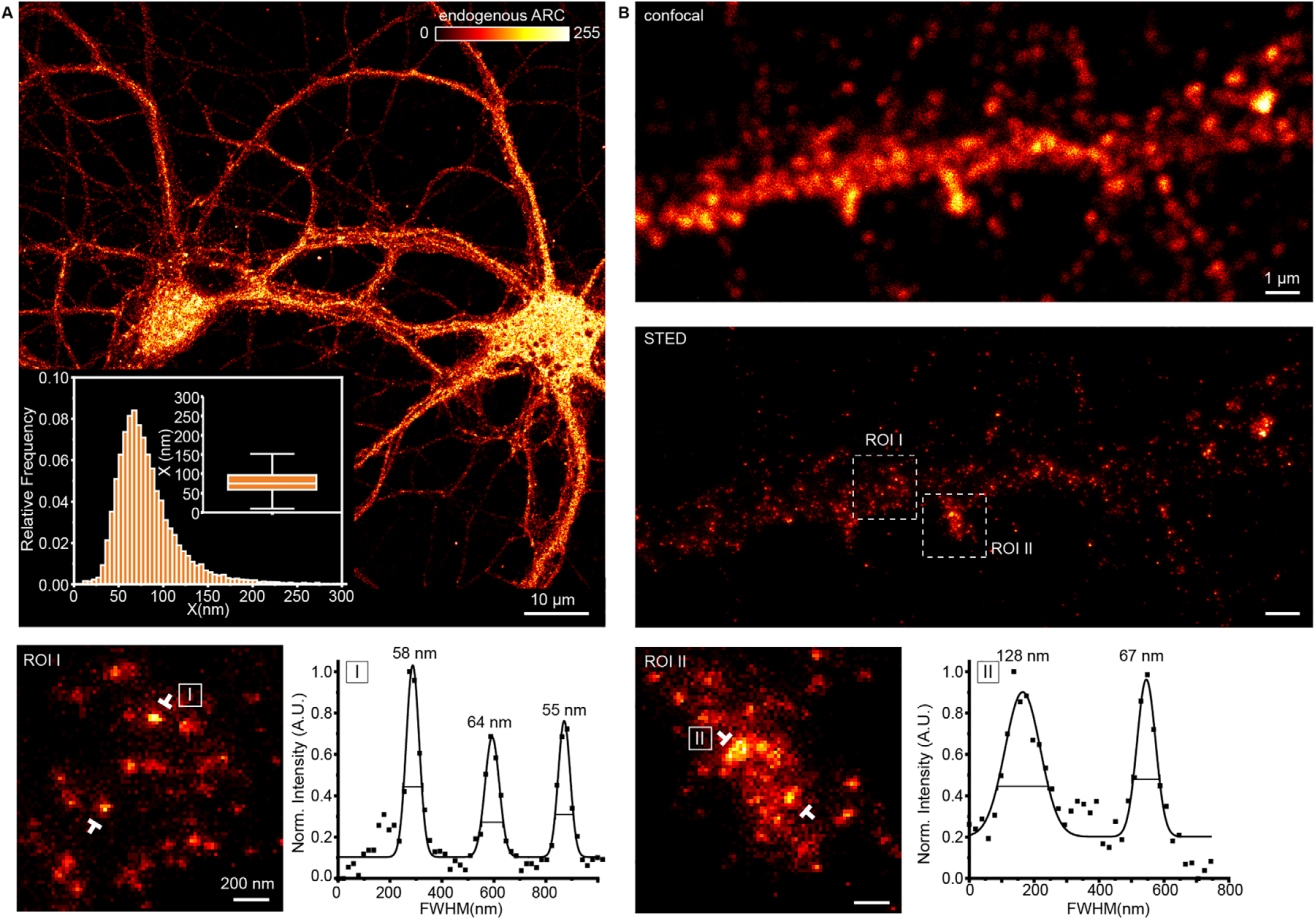
Nanoscale organization of ARC in primary neuronal cultures.(A) Example of a primary cortical neuron (DIV22) where the protein ARC was immunostained and imaged with confocal microscopy. (Inset) Histogram reports the distribution of FWHMs of ARC nanoclusters obtained from 26 neurons (DIV20–22) derived from 3 independent cultures. (B) Example of a neurite (DIV22) where ARC was imaged with confocal (top) and with STED (bottom) microscopy. In ROI I, ARC nanoclusters of 58, 64 and 55 nm FWHM are measured in the dendritic shaft. In ROI II, ARC nanoclusters of respectively 128 and 67 nm FWHM are measured in the dendritic spine. Line profiles are traced between the white arrows.

Previous studies have reported the presence of ARC molecules in several neuronal compartments, including the nucleus, soma, dendritic shafts and spines [7, 29, 30]. In accordance with that, when we imaged endogenous ARC in mature cortical neurons (DIV20‐22) with confocal resolution, we detected fluorescence distributed all over the cytosol (Figure 1A), with brighter puncta in spines and a highly variable expression level from neuron to neuron (Figure S1A). To access the finer spatial organization of ARC within the packed neuronal compartments, we used super‐resolution microscopy. STED microscopy unveiled the presence of a much smaller cluster organization, with clusters ranging from 30 to 300 nm in FWHM and distributed across the neurons including processes and spines (Figure 1A inset, B).

**FIGURE 2:**
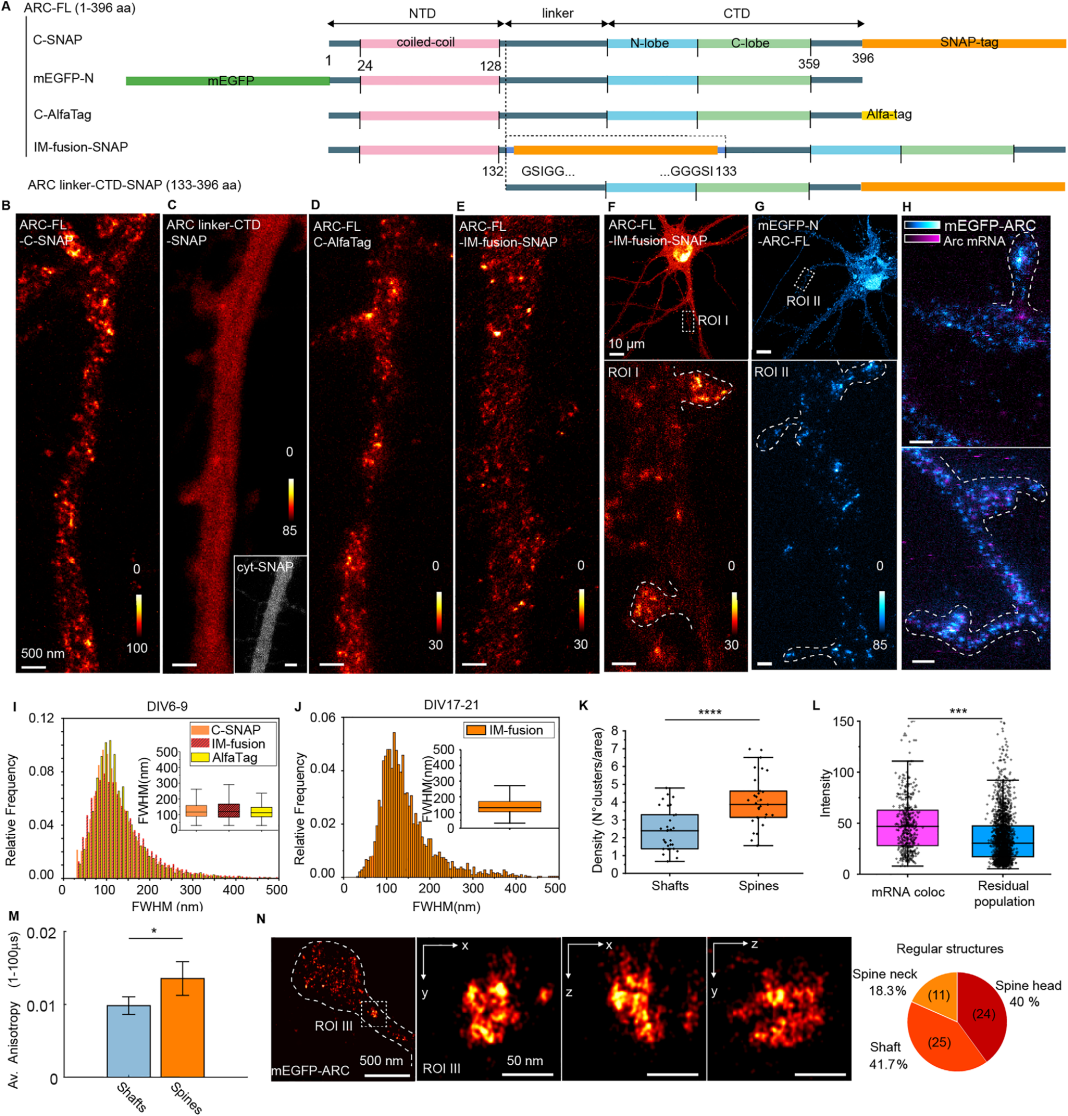
ARC is organized in nanoclusters in primary neuronal cultures. (A) Schematic representation of the ARC tagging strategies (SNAP‐tag, orange; AlfaTag, yellow; mEGFP, green) and positioning (N‐term, C‐term, IM‐fusion) used in the work for ARC‐FL (1–396 aa) and linker‐CTD truncated mutant (133–396 aa). ARC domains are in different colors (NTD, pink; flexible linker, black; N‐lobe, blue; C‐lobe, green). (B) Example of a cortical neurite (DIV6–9) expressing ARC‐FL‐C‐SNAP in live STED microscopy. (C) Example of a cortical neurite (DIV6–9) expressing ARC linker‐CTD‐SNAP, where no nanoclusters could be identified in live‐cell STED microscopy, similar to cytosolic SNAP (cyt‐SNAP, inset). (D) Example of a neurite (DIV7) expressing ARC‐FL‐C‐AlfaTag in STED microscopy. (E) Example of a cortical neurite (DIV6–9) expressing ARC‐FL‐IM‐fusion‐SNAP where the tag is placed as intramolecular fusion (IM‐fusion) between the NTD and the flexible linker in homology with human HIV‐1 GAG protein. (F) Mature primary hippocampal neuron (DIV21) expressing ARC‐FL‐IM‐fusion‐SNAP. In ROI I ARC nanoclusters show localization in the dendritic shaft and spine head. (G) Mature primary hippocampal neuron (DIV21) expressing mEGFP‐N‐ARC‐FL. In ROI II, ARC nanoclusters show localization in the dendritic shaft and spine head. (H) Mature primary cortical neuron (DIV20) expressing mEGFP‐N‐ARC‐FL (cyan) where Arc mRNA (magenta) was labelled via RNA FISH. A subset of ARC nanoclusters is shown to colocalize with the mRNA. (I) The histogram reports ARC nanocluster FWHM distributions from three labelling strategies. ARC‐FL‐C‐SNAP (22 DIV6–9 cortical neurons from 5 independent cultures, N = 24357 fitted nanoclusters). ARC‐FL‐IM‐fusion‐SNAP (13 DIV6–9 cortical neurons from 3 independent cultures, N = 12429 fitted nanoclusters). ARC‐FL‐C‐AlfaTag (10 DIV7 hippocampal neurons, N = 4282 fitted nanoclusters). As an inset, the box plot reports the difference in the ARC nanocluster FWHM distribution for three labelling strategies. (J) The histogram reports ARC‐FL‐IM‐fusion‐SNAP nanocluster FWHM distribution (18 (DIV17–21 cortical neurons from 5 samples in 2 independent cultures, N = 24357 fitted nanoclusters). (K) The box plot represents the significantly higher density of ARC nanoclusters in spines (orange) versus dendritic shaft (blue). Each data point is the average density value per spines and shaft per image (18 DIV17–21 cortical neurons from 5 samples in 2 independent cultures, N = 24357 fitted nanoclusters, two‐sample two‐sided Student's t test p‐value = 1.11×10^−4^, box plots show the 25–75% interquartile range, with the middle line representing the mean, and the whiskers derived from 1.5 * interquartile range). (L) Box plots showing the significantly higher intensity of ARC nanoclusters in colocalization with Arc mRNA (two‐sample two‐sided Student's t test p‐value = 1.81×10^−15^). Data are obtained from 4 cortical neurons (DIV20) (N = 1075 fitted nanocluster). Box plots show the 25–75% interquartile range, with the middle line representing the mean, and the whiskers derived from 1.5 * interquartile range). (M) Average fluorescence anisotropy in the time window 1–100 µs measured with STARSS for ARC‐IM‐fusion‐rsEGFP2 in spines (orange) and in shafts (blue) from 3 independent cultures in four sessions in different days. Shaft anisotropy from 50 FOVs: 0.0061± 0.0020 CI 95%, median: 0.005, quartiles: 0.003–0.009, whiskers: ‐0.006–0.016; spines anisotropy from 16 FOVs: 0.0139 ± 0.0072 CI 95%, median: 0.0099, quartiles: 0.003–0.026, whiskers: ‐0.0056–0.034; T‐test p‐value = 0.003. (N) (left panels) Example of a dendritic spine from cortical neurons (DIV22) expressing mEGFP‐N‐ARC‐FL imaged with 3D DNA‐PAINT. ROI III shows an example of ARC high‐order assemblies with radially symmetric organization shown in x‐y, x‐z, and y‐z orientation. (right panel) Pie chart showing the localization of 60 ARC high‐order assemblies (21 neurons from 2 independent cultures) localized in the head of dendritic spines (40%, N = 24), in the spine neck (18.3%, N = 11) and in dendritic shafts (41.7%, N = 25).

Since the presence of clusters could be induced by the polyclonal nature of antibodies [31] or affected by fixation [32], we developed additional labeling strategies for ARC that are compatible with both live and fixed super resolution imaging. Considering that the steric hindrance of the tag might impact ARC oligomerization, we a) tested tags of different sizes and linked them to different ARC protein domains (Figure 2A and b) maintained a subpopulation of untagged ARC.

To visualize ARC in live neurons, ARC full‐length (ARC‐FL) was tagged with a SNAP‐tag [33] at the protein C‐terminal (ARC‐FL‐C‐SNAP) (Figure 2B), or between its N‐terminal domain (NTD) and the flexible linker connecting to the C‐terminal domain (CTD) (ARC‐FL‐IM‐fusion‐SNAP) [26] (Figure 2E). The second construct was specifically designed based on the homology of ARC with retroviral GAG polyprotein to minimize the steric hindrance of the tag and it was previously shown to preserve GAG‐like oligomerization properties when co‐expressed with the protein [26]. SNAP‐tag is optimal for live‐cell STED imaging since SNAP‐ligands can be coupled with highly permeable and photostable fluorescent dyes. Live STED imaging confirmed the data from fixed neurons, showing ARC neuronal localization in the nucleus (Figure S2A) and processes, and its spatial organization in nanosized clusters with a distribution ranging from 30 to 300 nm (Figure 2I).

To test the influence of the tag size on the cluster formation, ARC‐FL was also tagged with the short peptide AlfaTag [34] (ARC‐FL‐C‐AlfaTag, Figure 2D) and immunostained with anti‐AlfaTag nanobodies or with mEGFP (mEGFP‐N‐ARC‐FL [29]^,^, Figure 2G) and immunostained by anti‐EGFP nanobodies to reach higher labeling densities than with anti‐AlfaTag nanobodies [35]. The last approach is preferable for super‐resolution imaging techniques such as DNA‐PAINT, where high localization accuracy needs to be coupled with a dense labeling strategy to achieve an accurate nanoscale representation of the labelled ARC.

Importantly, for all the constructs, the ARC coding sequence was flanked by Arc 5’‐ and 3’‐ UTRs given their relevance for ARC local translation. We evaluated the different tagging strategies first in early developmental stage (DIV6–9) rat primary cortical neurons where the minimal to null endogenous expression of ARC [30, 36] limits the observation mostly to the labelled protein. STED images of the different tagging methods, in both fixed and live neurons, showed both comparable neuronal localization and cluster size distributions, peaking at 95–105 nm (Figure 2I).

Interestingly, expression of a truncated ARC mutant lacking the N‐terminal domain (NTD) (ARC linker‐CTD‐SNAP) dramatically affects the spatial organization of ARC. STED images of ARC linker‐CTD revealed a homogenous cytosolic distribution, comparable to that of a SNAP‐tag volume staining, with no visible clusters (Figure 2C and inset). These results are consistent with previous studies identifying the NTD as the domain required for ARC oligomerization [16, 19]. Moreover, because this mutant retains binding sites for ARC interaction partners, such as AP2 and Dynamin‐2 as well as for synaptic partners such as TARPγ2 and CaMKII, we conclude that the NTD alone is sufficient for nanocluster formation and likely also for ARC oligomerization.

To investigate the localization of ARC nanoclusters in mature primary cortical and hippocampal neurons (DIV17–23, Figure 2F), we imaged ARC‐FL‐IM‐fusion‐SNAP with STED microscopy. ARC nanoclusters were observed in both dendritic shafts and spines, with a higher density in spines (Figure 2K), and exhibited a size distribution peaking at 117 nm (Figure 2J), comparable to that observed in fixed samples (Figure 2G; Figure S2C,D). A small population (23.2% of the total) of ARC nanoclusters was found in spatial proximity with Arc mRNA, detected with RNA FISH. These nanoclusters were significantly brighter than the residual population (Figure 2H, L), and they were found also in the heads of the dendritic spines.

To complement the super resolution imaging data in the investigation of ARC nanoclusters, we used STARSS [26], a spectroscopic method which couples time‐resolved fluorescence anisotropy (TR‐FA) to photo‐switching and thus extends the investigation of molecular assemblies to arbitrary sizes. Rotational diffusivity measurements are less sensitive to labeling densities as long as the fluorescent reporter is rigidly connected to the molecule of interest; only a few fluorescent molecules are representative of the global motion of a cluster.

In neurons expressing ARC‐IM‐fusion‐rsEGFP2 (DIV16‐17), the average anisotropy in the time window 1–100 µs was higher in spines compared to dendrites (Figure 2M), suggesting that ARC tended to form larger nanostructures in spines. The observed anisotropy decay, averaging from 0.014–0.006 within the observed temporal window, is consistent with multiple ARC assemblies: a fast diffusive population of monomers or low‐order assemblies; a fraction of ARC (∼15% of the total population of fluorescently labelled proteins) forming structures with effective hydrodynamic sizes of ∼ 60–90 nm; and a smaller fraction associated with larger assemblies (greater than 200 nm) or immobile structures (Figure S2E).

Both STARSS and STED data confirm the presence of ARC nanoclusters in the synaptic compartment of mature neurons.

Given the broad size distribution of ARC nanoclusters and their different assemblies observed with STED microscopy and STARSS anisotropy, we used 3D DNA‐PAINT to further examine their spatial sub‐organization. Primary neurons expressing mEGFP‐N‐ARC‐FL revealed distinct ARC assemblies: small ARC puncta (approaching the limit of localization accuracy, NeNA [37]: ∼ 5 nm) in the cytosol and spines (average diameter 19.7 nm, average volume 4.06×10^3^ nm^3^), corresponding to the fast‐diffusive monomeric or low‐order assemblies observed in STARSS; and ARC larger assemblies of ∼60–80 nm in diameter (average volume 24.6×10^4^ nm^3^) with a tendency toward radial symmetry (Figure 2N). These assemblies were preferentially localized in spines (40% in spine heads, 18.3% in spine necks) but were also present in shafts (41.7%; Figure 2N, right panel). By comparing their average volume with that of low‐order assemblies (Figure S2E, G), we estimate that each high‐order assembly comprises ∼60 copies of the low‐order unit. Assuming these low‐order units correspond primarily to monomers, we estimate that each high‐order assembly contains ∼60 ARC molecules. Although relatively rare (N = 60 in 21 neurons from 2 independent experiments), their occurrence did not correlate with mEGFP‐N‐ARC‐FL expression levels, indicating they were not artifacts of overexpression (Figure S2H). Considering the limited field of view (FOV) imaged per neuron (65 um^2^), we estimate an overall variability ranging from 0 to 240 high‐order assemblies per neuron.

Altogether, our data supports the conclusion that ARC assembles into distinct complexes which localize preferentially in dendritic spines.

### 2.2 ARC Nanoscale Organization at the Excitatory Synapse

**FIGURE 3:**
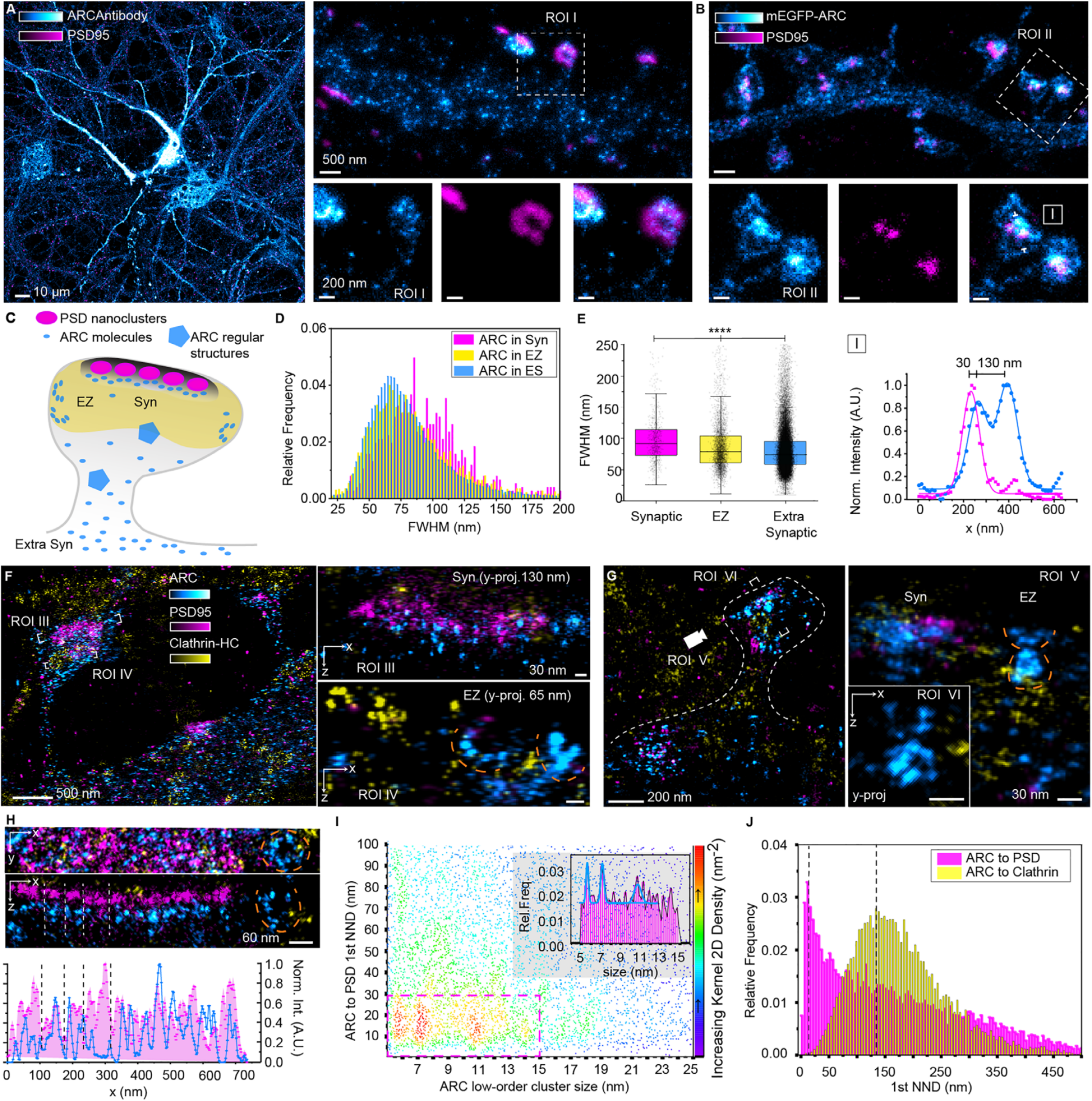
ARC nanoscale organization at the excitatory synapse. (A) Representative image of primary cortical neurons (DIV21) with ARC (cyan) and PSD‐95 (magenta) immunostained. ROI I shows close‐up of synapses where ARC colocalizes with PSD‐95. (B) Mature cortical neurite (DIV21) expressing mEGFP‐N‐Arc‐FL (cyan) under the control of ARC endogenous promoter and immunostained for PSD‐95 (magenta). In ROI II, ARC shows nanoclusters of different sizes localized in the head of the spine in proximity and co‐localizing with PSD‐95. (lower panel) Line profile traced along the white arrows. (C) The cartoon representation shows the different ARC subpopulations which localize within the peri‐synaptic level as observed in STED and DNA‐PAINT imaging. Refers to Table 1. (D) The histogram reports the distributions of ARC nanocluster sizes, measured in STED images, for ARC in co‐localization with PSD‐95 (Syn, magenta), within the endocytic zone (EZ, within 200 nm from PSD‐95 perimeter, yellow) and extrasynaptic (ES, blue) (26 neurons from 3 independent cultures). (E) The box plot shows the significant difference in nanocluster sizes between Synaptic ARC in co‐localization with PSD‐95 (Syn), ARC within the EZ (EZ, within 200 nm from PSD‐95 perimeter) and extra‐synaptic ARC (ES). Synaptic ARC median FWHM: 92 nm, EZ ARC median FWHM: 79 nm, extra‐synaptic ARC median FWHM: 73.5 nm. Box plots show the 25%–75% interquartile range, with the middle line representing the mean, and the whiskers derived from 1.5 * interquartile range. Two‐sample two‐sided Kolmogorov–Smirnov test p‐values respectively: 2.6934e‐4, 1.6397e‐17, 1.4041e‐18). Data derived from 28 neurons from three independent experiments in three independent cultures. (F) Example of a dendrite with synapses located in spine heads and in the shaft from DIV22 cortical neuron expressing mEGFP‐N‐ARC‐FL (cyan), immunostained for PSD‐95 (magenta) and Clathrin‐HC (yellow) and imaged with 3D DNA‐PAINT. ARC accumulates at the synaptic level (Syn) for certain synapses (ROI III) but not for others. At the EZ level, ARC semi‐circular organization can be observed (ROI IV, dashed orange lines). (G) Example of a dendritic spine from mEGFP‐N‐ARC‐FL expressing DIV22 cortical neuron (cyan), immunostained for PSD‐95 (magenta) and Clathrin‐HC (yellow) and imaged with 3D DNA‐PAINT. In ROI V, observed from the white camera icon direction, ARC is shown in accumulation at the synaptic level (Syn) and in semi‐circular and regular assemblies at the EZ level (EZ). ROI VI shows a 65 nm y‐projection of ARC regular structures perpendicularly sliced along white squared lines. (H) Example of a dendritic shaft synapse from DIV22 cortical neuron expressing mEGFP‐N‐ARC‐FL (cyan) and immunostained for PSD‐95 (magenta) and Clathrin‐HC (yellow) imaged with 3D DNA‐PAINT. Observing the synapse axially (130 nm y‐projection) ARC molecules are shown to partially align with PSD‐95 nanoclusters as reported in the line profile below. Next to PSD‐95, an example of ARC circular structure is observed and highlighted with a dashed orange line. (I) The scatter plot shows the correlation between ARC PSD‐95 1^st^ NNDs to ARC low‐order assemblies' size. The density color map reveals three higher density areas corresponding to subpopulations of ARC diameters of 5.7, 7.2 and 10.7 nm within 30 nm from PSD‐95. The frequency distribution in the inset reports the peaks corresponding to the three populations. (J) The histogram reports the distributions of 1^st^ NNDs from ARC to PSD‐95 (magenta), which peaks at 12.5 nm, and from ARC to Clathrin‐coated vesicles (yellow), which peaks at 132.5 nm. (13 neurons from 2 independent neuronal cultures).

Considering the role of ARC in the endocytosis of AMPA receptors [30], its formation of complexes with PSD‐95 [38], and its function as an actin stabilizer [13], we characterized endogenous ARC organization at both the synaptic and endocytic zone (EZ). EZ was defined as the area located 200 nm away from the PSD‐95 perimeter, consistent with previous ultrastructural and super‐resolution studies [39]^.^ ARC nanoclusters at the synaptic and EZ displayed a significantly increased apparent size and brightness as compared to extra‐synaptic ARC (Figure 3D, E; Figure S3.2A; synaptic ARC median FWHM: 92 nm, EZ ARC median FWHM: 79 nm, extra‐synaptic ARC median FWHM: 73.5 nm).

To rule out labeling artifact, we repeated the experiment expressing mEGFP‐N‐ARC‐FL under the control of ARC endogenous promoter, as previously reported by Okuno et al., 2012 [29]. STED imaging revealed ARC in close spatial proximity to the immunostained PSD‐95 (Figure 3B). Moreover, synaptic mEGFP‐N‐ARC‐FL nanoclusters displayed larger diameters and higher brightness compared with extra‐synaptic nanoclusters, with synaptic‐to‐extrasynaptic ratios of size and intensity comparable to those observed for endogenous ARC staining (Figure S3.1A–D). ARC is known to be rapidly expressed and localized in dendrites upon synaptic activation. However, how ARC nanoscale organization is altered upon induction of synaptic plasticity has never been addressed. To investigate that, we stimulated neurons with BDNF (4 h, 100 ng/ml), a neuropeptide known to trigger synaptic plasticity and local translation of ARC [1, 2, 31]. We observed a significant increase in ARC nanocluster density near the synaptic marker PSD‐95 (Figure S3.1E, F), together with a significant increase in size of both synaptic (Syn) and extra‐synaptic (ES) ARC nanoclusters (Figure S3.1E, G, H).

Altogether, our super‐resolution imaging data reveals an ARC organization in nanoclusters, whose size and synaptic density increases upon plasticity induction.

To further investigate the potential suborganization of ARC nanoclusters at the synaptic site, we performed 3D DNA‐PAINT. The expression of mEGFP‐N‐ARC‐FL was followed by immunostaining of PSD‐95 and Clathrin heavy chain (Clathrin‐HC), which was used as an EZ marker (Figure 3F). A comprehensive analysis of the data allowed the identification of distinct ARC populations at both the synaptic and EZ level: ARC molecules as low‐order assemblies were denser in the surroundings of PSD‐95, and regular and semi‐circular organizations of ARC were located laterally to the PSD, extending into the EZ (Figure 3C, F, G). Focusing on ARC low‐order assemblies at the PSD level, we found that the majority (52 out of 81) of analyzed synapses contained at least 30% more ARC molecules in colocalization with PSD‐95 than in extra‐synaptic areas. Furthermore, by measuring the distance from each ARC low‐order assembly to the first PSD‐95 nearest neighbor (1^st^ NND), we showed that ARC can be as close as the localization accuracy limit to PSD‐95 within a distribution that peaks at 12.5 nm (Figure 3J, magenta). The axial localizations of ARC molecules revealed distinct sub‐distributions: in 55% of analyzed synapses ARC molecules aligned to PSD‐95 (Figure 3H). Correlating the size of each ARC low‐order assembly to its 1^st^ PSD‐95 NN, we identified three distinct ARC size subpopulations of 5.7, 7.2 and 10.6 nm, located within 30 nm from PSD‐95 (Figure 3I).

Examining ARC lateral to the PSD within the EZ, it showed a semi‐circular organization relatively distant from Clathrin‐coated vesicles. Based on the number of ARC localizations in low‐order assemblies, we estimated that these ARC semi‐circles were constituted by 3 to 8 ARC units. In a minority of cases (23%), semi‐circular ARC organizations co‐existed at the same synaptic terminal with high‐order ARC assemblies, at a variable distance from 36 to 245 nm (Figure S3.2D). By measuring the 1^st^ NND from low‐order oligomers of ARC to Clathrin‐coated vesicles, we showed that ARC molecules are not found in colocalization with Clathrin‐coated vesicles but can be as close as 7.5 nm from them, with a distribution peak at 132.5 nm (Figure 3J). However, differently from PSD‐95, the correlation of the size of low‐order assemblies of ARC to Clathrin‐coated vesicle 1^st^ NND revealed multiple subpopulations of ARC located between 100 to 200 nm (Figure S3.2B). This suggests a higher complexity of ARC interactions further away from Clathrin‐coated vesicles. The broadness of the distribution might result from considering ARC molecules located both at PSD and EZ levels. In the attempt to limit the observation to ARC molecules at the EZ level, we calculated the 1^st^ NND from Clathrin‐coated vesicles to ARC (Figure S3.2C). In this case, the distance distribution peaked at 75 nm.

PAINT images of ARC and the actin cytoskeleton show no clear colocalization of high‐order assemblies of ARC and the synaptic actin bundles (Figure S3.2E).

Consistently, STED imaging of ARC following treatment with BDNF stimulation reports a significant increase in the apparent size of both synaptic and extra‐synaptic ARC nanoclusters (Figure S3.1E‐H), potentially reflecting the accumulation of ARC low‐order assemblies interacting with actin‐binding proteins within dendritic spines during LTP.

To directly test this possibility, we performed DNA‐PAINT on BDNF‐stimulated neurons expressing mEGFP‐N‐ARC and measured the 1st NND between ARC low‐order assemblies and individual PSD‐95 molecules (Figure S3.2F, G). Notably, ARC low‐order assemblies were located further from PSD‐95 following BDNF stimulation, suggesting that this population may contribute more to spine stabilization than to direct interactions with post‐synaptic density proteins.

Overall, our 3D DNA‐PAINT data reveal distinct ARC populations among synaptic ARC nanoclusters, including low‐order assemblies within the PSD, semi‐circular organization at the EZ, and higher‐order assemblies enriched in dendritic spines (Table 1).

**TABLE 1:** ARC subpopulations identified at the peri‐synaptic level. The table summarizes the different types of ARC organization identified with DNA‐PAINT at the peri‐synaptic level of neuronal cells. Refers to Figure 3.

Population name	Average size	Morphology	Localization
Low‐order assemblies	19.7 nm diameter	Single molecules	Cytosolic
Low‐order assemblies	5.7, 7.2 or 10.6 nm diameter	Single molecules aligned with PSD‐95	Synaptic zone, ≤30 nm from PSD‐95
High‐order assemblies	∼60–80 nm diameter, ∼60 low‐order assemblies	Ordered and radially symmetric organization	Spine heads and necks, lateral to the PSD‐95, extending into the EZ
Semi‐circular organizations	∼30–100 nm, ∼3 –8 single molecules	Semi‐circular / curved structures	Within EZ, not co‐localized with PSD‐95

### 2.3 ARC NTD is Required for ARC co‐Localization with AMPA Receptors

Super‐resolution imaging and STARSS anisotropy demonstrated the coexistence of different ARC populations within primary neurons. This suggests a potential differential involvement in ARC functions. ARC is known for playing a role in the endocytosis of AMPARs by binding the AMPAR accessory subunit TARPγ2 with its CTD (N‐lobe) [40, 41]. Given the established role of ARC in AMPAR endocytosis, we next asked whether specific ARC nanoscale organizations are associated with synaptic AMPAR nanoclusters [42, 43, 44].

**FIGURE 4:**
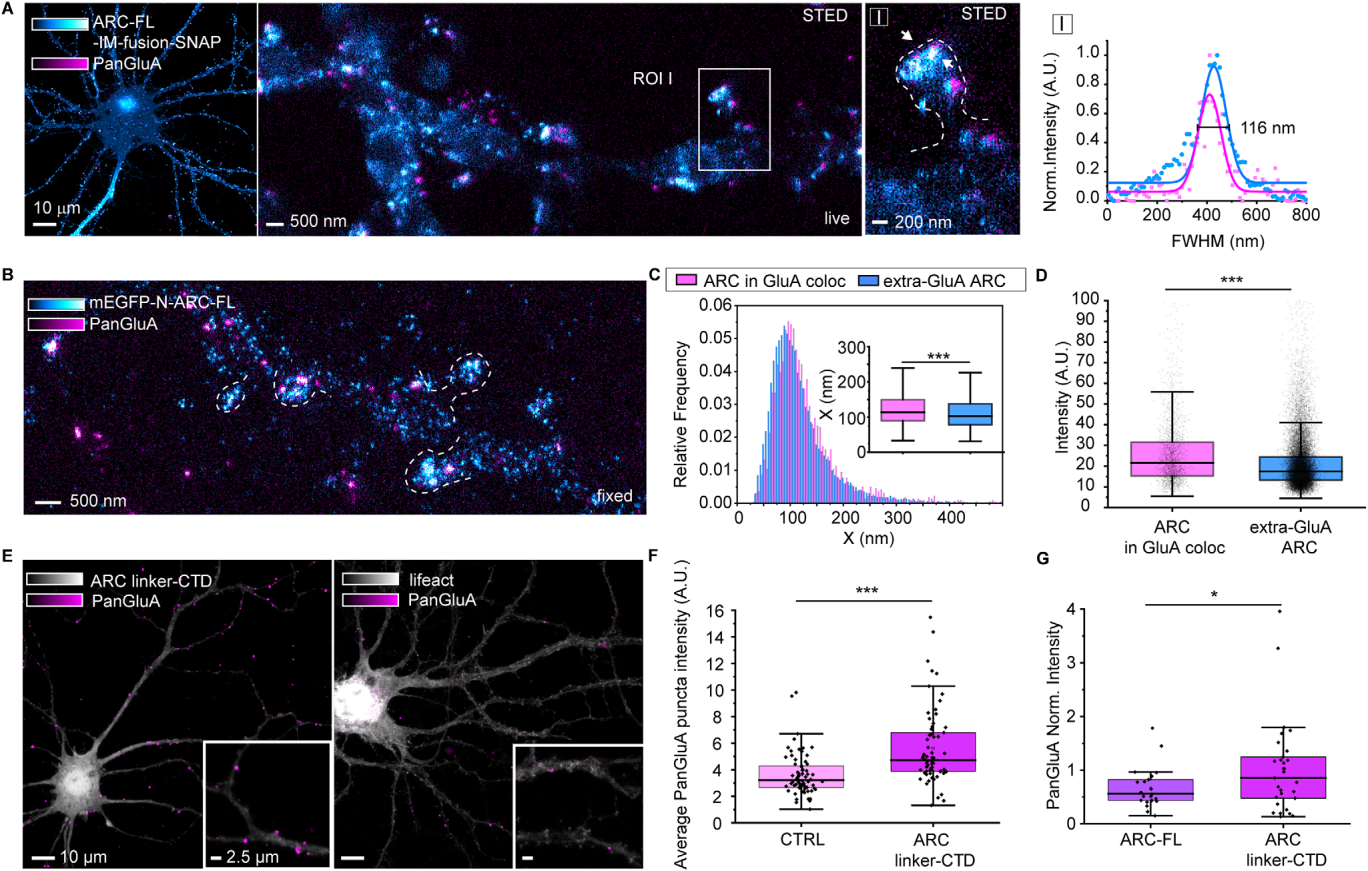
ARC linker‐CTD increases the surface level of AMPA receptors. (A) Representative primary cortical neuron (DIV20) genetically encoding ARC‐FL‐IM‐fusion‐SNAP (cyan) and live immunostained for PanGluA (magenta). In ROI I, ARC nanoclusters of different sizes localize in spine heads in proximity and in colocalization with AMPA receptors. ARC nanoclusters of 116 nm FWHM is shown to co‐localize with GluA, the line profile is traced along the white arrows. (B) Representative mature hippocampal neurite (DIV21) expressing mEGFP‐N‐ARC‐FL (cyan) and PanGluA (magenta), live imunostained prior to fixation. (C) The histogram reports the FWHM distributions of mEGFP‐N‐ARC‐FL nanoclusters co‐localizing with GluA (pink) and the extra‐GluA (blue) population (24 DIV21–22 primary hippocampal neurons from 2 independent cultures, N = 25080 fitted nanoclusters). As a histogram insert, the box plot shows that ARC nanoclusters in colocalization with GluA are significantly bigger than extra‐GluA ones. Box plots show the 25%–75% interquartile range, with the middle line representing the mean, and the whiskers derived from 1.5×interquartile range. Two‐sample two‐sided Kolmogorov–Smirnov test p‐value: 1.61×10^−25^). (D) The box plot shows that ARC nanoclusters co‐localizing with GluA (pink) are significantly brighter than extra‐GluA ones (blue). Box plots show the 25%–75% interquartile range, with the middle line representing the mean, and the whiskers derived from 1.5×interquartile range. Two‐sample two‐sided Kolmogorov–Smirnov test p‐values: 3.37×10^−5^). (E) Representative primary cortical neurons (DIV20–21) expressing ARC linker‐CTD‐SNAP and Lifeact‐YFP (gray) and live immunostained for GluA receptors (magenta) where GluA receptors puncta intensities can be compared. (F) The box plot shows that GluA puncta intensities are significantly higher for ARC linker‐CTD‐SNAP expressing neurons compared to Lifeact‐YFP expressing (CTRL) neurons. Each data point represents the average AMPA puncta intensity per neuron (N_linker‐CTD_ = 66, N_CTRL_ = 70, from 3 independent primary cortical cultures). Box plots show the 25%–75% interquartile range, with the middle line representing the mean, and the whiskers derived from 1.5×interquartile range. Two‐sample two‐sided Kolmogorov–Smirnov test p‐value: 7.25×10^−8^. (G) The box plot shows that the PanGluA normalized by the level of the construct expression is significantly higher for ARC linker‐CTD‐SNAP expressing neurons than for ARC‐FL‐SNAP. Each data point represents the average AMPA puncta intensity per neuron divided by ARC intensity (N_linker‐CTD_ = 29, N_Arc‐FL_ = 22, from 2 independent primary cortical cultures). Box plots show the 25%–75% interquartile range, with the middle line representing the mean, and the whiskers derived from 1.5×interquartile range. Two‐sample two‐sided Kolmogorov–Smirnov test p‐value: 0.0307.

To address this question, we first expressed ARC‐FL‐IM‐fusion‐SNAP in neurons and evaluated its proximity to GluA with live STED microscopy. Consistent with the known interaction of ARC with AMPAR complexes, we observed close spatial association between ARC nanoclusters and GluA at the synaptic sites (Figure 4A; Figure S4.1). We further characterized this association in fixed neurons expressing mEGFP‐N‐ARC‐FL and imaged together with GluA (Figure 4B). ARC nanoclusters colocalizing with GluA displayed a significantly increased apparent size and brightness (Figure 4D, C) (colocalizing median FWHMxy: 113.5 nm, residual population median FWHMxy: 102.5 nm) than the extra‐GluA components. To exclude that large nanoclusters could derive from the 3D spine geometry projection, we took advantage of 3D STED microscopy, which features an axial resolution of about 70 nm [45], and confirmed that they indeed colocalized with AMPAR (Figure S4.2A). The observed colocalization might result from the binding of ARC with its post‐synaptic interaction partner.

As shown with 3D DNA‐PAINT, ARC nanoclusters at the synaptic level were predominantly constituted by low‐order species. However, we were unable to clearly differentiate between distinct types of low‐order assemblies. To disclose the oligomeric state of this ARC population, the truncated mutant ARC linker‐CTD‐SNAP, which preserves the binding site for most of its known interacting partners (AP2, Dynamin‐2, TARPy2 and CaMKIIa) but does not form oligomers (Figure 2C), was expressed and imaged together with GluA in live neurons with STED microscopy (Figure S4.2B). No ARC nanoclusters were found colocalizing with AMPA receptors. These data indicate that the ARC NTD is necessary for the formation of ARC nanoclusters associated with AMPARs, suggesting that ARC oligomerization, rather than monomeric ARC alone, is required for stable synaptic association with AMPARs.

In addition, in neurons expressing ARC linker‐CTD‐SNAP, GluA puncta were significantly brighter than those measured in control conditions, where neurons were expressing Lifeact‐YFP (average norm. intensity CTRL: 3.064; average norm. intensity ARC linker‐CTD‐SNAP: 3.854) (Figure 4E, F) or ARC‐FL‐SNAP (Figure 4G). No difference was observed in the total number of GluA puncta (Figure S4.2C). These observations indicate that the expression of ARC linker‐CTD affects AMPA receptor surface levels consistent with a dominant negative‐like effect on endogenous ARC assemblies and their relative function. Therefore, we conclude that the ARC NTD is critical for synaptic association and for processes regulating AMPAR surface levels, consistent with its role in AMPAR endocytosis.

### 2.4 ARC‐Membrane Interaction Via its NTD is Modulated by Palmitoylation and PIP Lipid Interaction

So far, we have shown the requirement of the ARC NTD for the organization of ARC in synaptic nanoclusters, which is consistent with the presence of ARC oligomerization‐dependent assemblies at the synaptic level. Together with that, we also used 3D DNA‐PAINT to identify semi‐circular organization of ARC at the EZ. This population might represent ARC molecules involved in endocytic events [7, 46] given their spatial organization and localization relative to the EZ. It has been previously suggested that ARC might interact directly with lipid membranes via its NTD [46, 47, 48], which can be palmitoylated to promote AMPAR endocytosis. However, ARC‐membrane interactions have not been substantially explored in this context.

**FIGURE 5:**
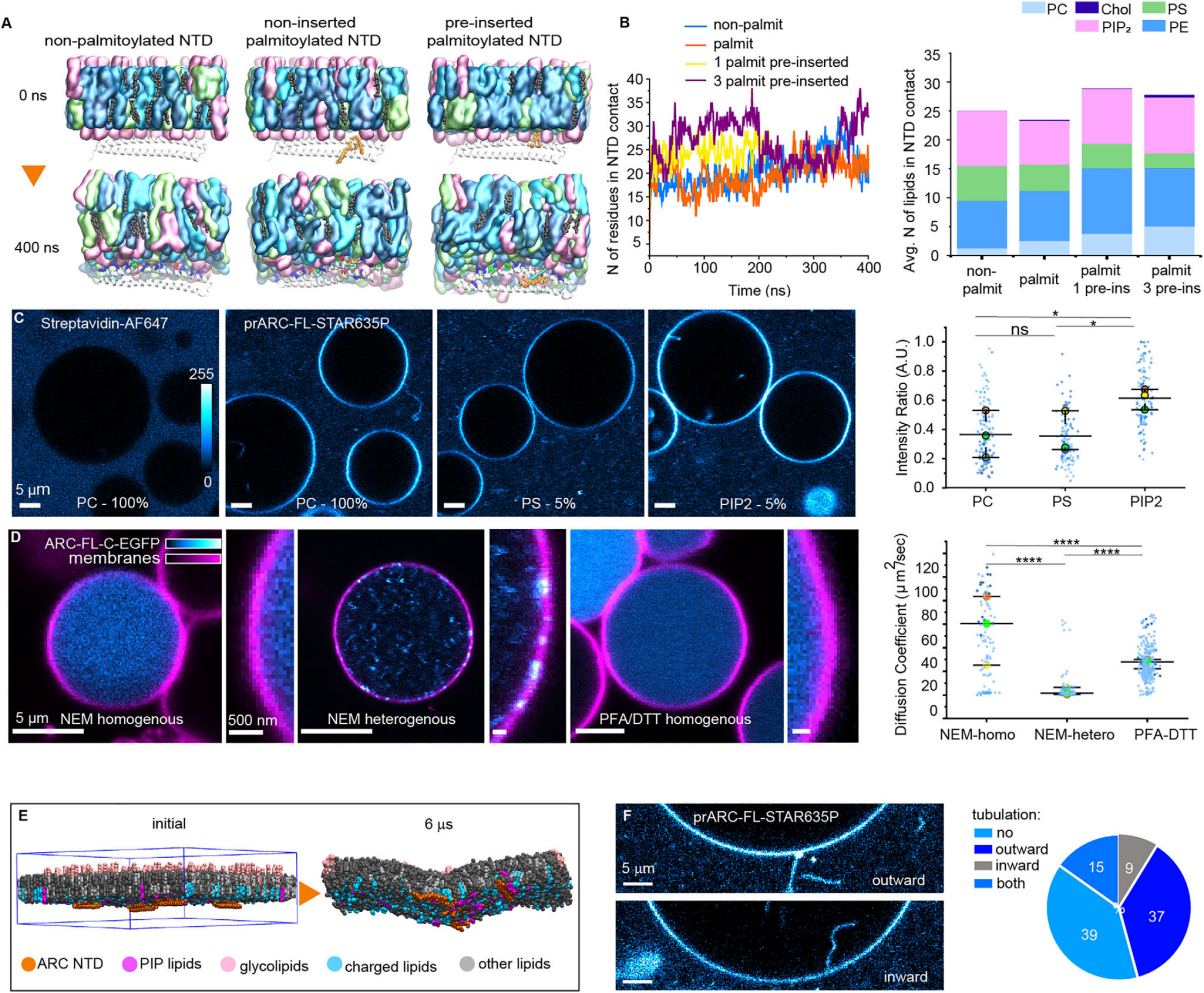
Palmitoylation and PIP lipids modulate, but they are not required to mediate ARC‐membrane interaction. (A) Atomistic simulation of ARC‐NTD (24–134 aa)‐membrane interaction. ARC‐NTDs are placed in proximity to lipid bilayers composed of PE, PS, PC, PIP2 lipids and cholesterol. ARC NTDs are respectively non‐palmitoylated, triple palmitoylated but not pre‐inserted in lipid bilayer or triple palmitoylated and pre‐inserted. Simulation duration: 400 nsec. (B) (left panel) Graph showing the number of ARC‐NTD residues in contact with lipids over the time of the simulation for the different conditions. (right panel) Graphs showing the average number of lipids per type in contact with ARC‐NTDs for the different conditions. (C) (left panels) GUVs incubated with streptavidin‐AF647 or rat prARC‐FL‐STAR635P show ARC interaction with lipid bilayers for distinct GUV lipid composition (100% PC, 95% PC + 5% PS or 95% PC + 5% PIP2). (right panel) Plot showing the ratio between ARC intensity at GUVs and ARC intensity in PBS solution for the three GUV compositions. PIP2 GUVs ARC intensity ratio is significantly higher than in PC and PS conditions. The plots’ the middle line represents the mean, and the whiskers are derived from 1.5×interquartile range. Two‐sample two‐sided Kolmogorov–Smirnov test p‐values: PC–PS p‐value 0.97, PS–PIP2 p‐value 0.0326, PC–PIP2 p‐value 0.0326, from 3 independent experiments whose mean values are color‐coded). (D) (left panel) Representative images of HeLa cells ARC‐FL‐C‐EGFP‐derived GPMVs. ARC‐FL‐C‐EGFP is represented in cyan and the plasma membrane, labelled with red dye conjugated phospholipids, is in magenta. Two phenotypes were isolated from NEM derived GPMVs, homogeneous (N = 27) and heterogeneous (N = 44), while for DTT‐derived vesicles only homogeneous phenotype was observed (N = 68). (right panel) FCS plot on GPMVs populations showing significantly different distributions of D_t_s (mean Dt_NEM‐ homogeneous:_ 41.5 um^2^/s, mean Dt_NEM‐ heterogeneous_: 4.8 µm^2^/s, mean Dt_PFA/DTT_: 25.6 um^2^/s, obtained from 3 independent experiments whose mean values are color‐coded. The plots show the middle line representing the mean, and the whiskers are derived from 1.5×interquartile range. Two‐sample two‐sided Kolmogorov–Smirnov test p‐values: PFA/DTT‐NEM_homo p‐value: 2.16×10^−10^, PFA/DTT‐NEM_hetero p‐value 9.49×10^−64^, NEM_homo‐NEM_hetero p‐value 8.97×10^−21^). (E) Snapshots from a coarse‐grained molecular dynamic simulation of 4 triple palmitoylated ARC‐NTDs inserted into a membrane mimicking the asymmetric lipid composition of the plasma membrane. (F) (left panel) Representative images of PC GUVs incubated with prARC‐FL‐STAR635P showing inward and outward tubulation. (right panel) Pie chart showing inward and outward tubulation percentages. Among the analyzed GUVs, 39% (N = 116) presented no tubulation, 37% (N = 110) had outward tubulation, 15% (N = 45) had both inward and outward tubulation while 9% (N = 26) showed inward tubulation.

To investigate whether the ARC NTD interacts directly with lipids and whether palmitoyl groups play a fundamental role in the process, we took advantage of atomistic molecular dynamic simulations. Since the structure of ARC NTD has been experimentally determined only at low resolution with small‐angle X‐ray scattering (SAXS) [24], we first used the deep learning methods TrRosetta [49] and AlphaFold [50] to predict the structure of ARC‐NTD (24‐134 aa) (Figure S5.1A). We obtained a prediction consistent with the low‐resolution SAXS structure, showing two alpha helices between amino acids 26 and 130 (TrRosetta) (Figure S5.1B, A) or 34 and 133 (AlphaFold) (Figure S5.1B, A). Next, four NTDs (non‐palmitoylated, palmitoylated, palmitoylated with 1 or 3 carboxylic tails pre‐inserted) were positioned next to a simulated lipid bilayer, which mimicked the physiological lipid composition of the inner plasma membrane leaflet (Figure 5A). The number of residues in contact with lipids rapidly increased within nanoseconds after the simulation onset and stabilized at values between 15 and 30, regardless of the NTD variant. This suggested that ARC NTD can, even in the absence of palmitoylation, strongly interact with lipids (Figure 5B, left panel). From the simulation, phosphatidyl‐ethanolamine (PE) and phosphatidylinositol 4,5‐bisphosphate (PIP2) were the lipids most in contact with the NTDs (Figure 5B, right panel). The negatively charged PIP2 lipids formed the majority of contacts, despite their considerably lower abundance (10 mol%) compared to PE lipids (40 mol%). Interestingly, phosphatidyl‐serine (PS) lipids made fewer contacts than PIP2, despite being more abundant (15 mol%) and negatively charged. This observation suggests that the presence of PIP2 lipids preferentially stabilizes the interaction between lipid membranes and the ARC NTD. In parallel, we also conducted the same type of simulations using a slightly different ARC NTD structure, predicted by ab initio modeling, using the Robetta server. The result gave a structure very similar to the one obtained with the TrRosetta model (Figure S5.1E), confirming that palmitoylation is not essential for ARC NTD‐membrane interaction (Figure S5.1D). Furthermore, coarse‐grained molecular dynamics simulation showed that lateral diffusion of NTDs along the lipid bilayer led to the accumulation of PIP lipids, corroborating the strong interaction between the NTD and PIP2 lipids (Figure S5.1C).

The simulations were experimentally supported by in vitro experiments with Giant Unilamellar Vesicles (GUVs). GUVs were incubated with non‐palmitoylated rat ARC protein purified from bacteria (prARC‐FL) and subsequently labelled with STAR635P dye (Figure 5C, left panel). prARC‐FL accumulated at the lipid bilayer of GUVs while Streptavidin‐Alexa647, used as a control, did not show any tendency to interact with lipids. As an additional control, we incubated GUVs solely with either NHS‐STAR635P dye or EGFP protein, and again no GUV membrane localization was observed (Figure S5.2B, C). These results showed that pr‐ARC‐FL can directly associate with lipid membranes, independently of palmitoylation, and without additional interaction partners.

To experimentally assess the effect of negatively charged lipids on ARC‐membrane association, the lipid composition of the GUVs was modified (Figure 5C, central panels). Consistent with the simulation, 5% PIP2 lipids in 95% phosphatidylcholine (PC) were enough to significantly increase the ratio between ARC at the membrane and in solution, while 5% PS did not result in the same effect (Figure 5C, right panel). Altogether these results showed the capability of ARC to interact with lipids and preferentially with PIP2 in an in vitro system, confirming the results obtained with the simulations.

prARC‐FL purified from bacteria intrinsically lacks palmitoylation, and GUVs do not replicate exactly the membrane composition of a cellular environment. Therefore, we took advantage of Giant Plasma Membrane Vesicles (GPMVs), a more complex system which allowed us to study the effect of palmitoylation on the ARC phenotype. GMPVs are vesicles derived from the cellular plasma membranes, and they lack PIP lipids, organelles, cortical actin and other cytoskeletal components, hence they can be used as a clean system to study membrane and protein interaction dynamics. In addition, the palmitoylation status can be modified using distinct vesiculation chemicals: NEM (N‐Ethylmaleimide) preserves palmitoylation while DTT (dithiothreitol) in combination with PFA (paraformaldehyde) removes the palmitoyl tails from proteins [51]. GPMVs were produced from HeLa cells expressing ARC‐FL‐C‐EGFP. The reasoning behind taking advantage of HeLa cells is that this cell type is reported to not significantly express neither ARC and Arc mRNA nor its synaptic interaction partners. Therefore, HeLa cells represent a heterologous expression system to evaluate potential ARC–ARC interaction behavior.

Two populations of GPMVs were obtained from NEM‐treated HeLa cells: GPMVs with ARC homogenously distributed or GPMVs with ARC organized in micrometric clusters and patches observed in proximity to the plasma membrane (Figure 5D, left and central panels). When PFA‐DTT was used, instead, only GPMVs with ARC homogenously distributed were observed. (Figure 5D, right panel).

We next took advantage of Fluorescence Correlation Spectroscopy (FCS) to investigate the ARC assembly states. In FCS data analysis, ARC assembly states were inferred from diffusion coefficients. For the NEM‐treated heterogeneous population, a broad distribution of states, from monomers to high‐order assemblies, was observed. For the NEM‐treated homogeneous population, the average diffusion coefficient of ARC (D_t_ = 41.5 µm^2^/s) was comparable with the estimated diffusion coefficient of the monomeric ARC‐FL‐C‐EGFP (D_t_ = 40.5 µm^2^/s). For DTT‐treated GPMVs, instead, the measured diffusion coefficient (D_t_ = 25.6 µm^2^/s) corresponded on average to the diffusion of an assembly which is 4.3 times slower than the monomeric ARC‐FL‐C‐EGFP. Additional experiments with the truncation mutant ARC linker‐CTD confirmed that neither NEM nor PFA‐DTT treatments induced artificial clustering (Figure S5.1F). These results indicate that the absence of PIP2 lipids does not abolish ARC membrane association. However, in DTT‐derived GPMVs, where neither PIP2 lipids nor palmitic anchors are present, no membrane recruitment was observed, and on the contrary the diffusion of an assemblies four times slower than the monomeric ARC was measured.

Experiments in GPMVs demonstrated that palmitoylation is important for ARC membrane localization. However, the GPMVs assay is not specific for ARC palmitoylation, but it might involve the palmitoylation of other proteins of the endocytic machinery, such as for example PICK1, which is known to interact with ARC itself [8]. Therefore, to directly assess the importance of ARC palmitoylation, we performed additional coarse‐grained molecular dynamics simulations. We considered palmitoylated and non‐palmitoylated NTDs positioned in solution close to a PIP‐free lipid bilayer (Figure S5.2A). We observed that palmitoylated NTDs interacted first with the membrane and ultimately with each other, whereas non‐palmitoylated NTDs interacted with each other first and eventually later with the membrane. These results suggest that, although palmitoylation is not essential for ARC‐membrane interaction, it increases the probability of ARC NTD to come in proximity to the membrane. Overall, our data indicate that ARC–lipid interactions can occur in the absence of palmitoylation and PIP lipids, however both of them affect the process by modulating the interaction probability and membrane recruitment of ARC in vitro and in cellular contexts.

To evaluate if these observations can be extended to neuronal membranes, GPMVs were produced from mEGFP‐N‐ARC expressing neurons. Similar phenotypes to HeLa cells were observed: GPMVs with homogenous ARC, and GPMVs with ARC organized in micrometric clusters and in proximity to the plasma membrane (Figure S5.2D).

Since we saw that ARC can interact with the membrane, to further investigate its potential direct involvement in endocytosis, we look at the potential ability of ARC to directly induce membrane bending. Mondal et al., 2020 [40] showed that Endophilin, one of the main proteins involved in endocytosis, binds plasma membranes, and it induces tubulation in GUVs. To test whether ARC might have a similar behavior, we first performed coarse‐grained simulations of ARC‐NTDs‐plasma membrane interaction in the presence of PIP lipids. The simulations indicate that ARC NTDs may increase local membrane curvature (Figure 5E), no matter if palmitoylated or not (Figure S5.2E). While the local curvature around clustered NTDs is predominantly facing inward, this causes an increase in outward curvature in surrounding membrane regions, which is especially evident in the absence of palmitoylation (Figure S5.2E, bottom panels). Moreover, incubating prARC‐FL‐STAR635P with GUVs resulted in the formation of inward and outward tubulation (Figure 5F, left panel). The tubules reached micrometers in length, the majority were facing outward (37% among the total GUVs analyzed), a minority (9%) were facing inward, and 15% of them showed both inward and outward tubulation (Figure 5F, right panel). No significant differences in ARC fluorescence intensities were observed for GUVs showing tubulation in any direction or GUVs with no tubulation (Figure S5.3B). Moreover, ARC intensity signal did not correlate with the number of tubules observed with confocal resolution (Figure S5.3C), suggesting that membrane deformations do not depend, at least at a macroscopic level, on ARC local concentration.

To assess the nanoscopic arrangement of ARC at the membrane in GUVs, we used 2D STED and observed membrane curvatures like what was observed in neurons with 3D DNA‐PAINT (Figure S5.3A). This corroborates the capability of ARC to directly interact with lipid bilayers and promote membrane deformation.

Our data reveal multiple ARC assemblies in neurons, including fast‐diffusing low‐order assemblies, synapse‐associated low‐order assemblies colocalizing with surface AMPARs, higher‐order assemblies (60–80 nm) predominantly localized in dendritic spines, and semi‐circular ARC organizations at the endocytic zone. In parallel, we show that ARC can directly associate with lipid bilayers and promote membrane curvature independently of additional protein interaction partners. Altogether, these findings support a model in which ARC oligomerization and ARC–membrane interactions contribute to its spatial organization at excitatory synapses and endocytic sites, providing a mechanistic framework for its role in AMPAR trafficking.

## 3 Discussion

Our work focuses on the synaptic protein ARC. For the first time, we provide a detailed characterization of its spatial organization at the subsynaptic level in relation to synaptic and EZ markers. Building on previous in vitro observations of ARC high‐order oligomers, we now analyze ARC assemblies in both live and fixed neuronal environments. Using super‐resolution microscopy and complementary spectroscopic techniques, we visualize ARC nanoclusters in neurons. These include both low‐ and high‐order assemblies, distinguished by structural (size and shape) and dynamic (translational and rotational) properties.

2D STED microscopy resolves ARC nanoclusters within the dendritic spines, resulting in a size distribution from 30 to 300 nm (Figures 1 and 2). STARSS anisotropy further confirms the presence of clusters with rotational diffusivity consistent with objects of ∼ 60–90 nm and > 200 nm in diameter (Figure 2). As seen with 3D DNA‐PAINT, across and near synaptic areas, ARC exists in diverse states: low‐order assemblies located beneath the PSD‐95 lattice, semi‐circular organizations at the endocytic zone, and radially symmetric ∼ 60–90 nm assemblies distributed throughout the spine (Figure 3) [28]. Following treatment with BDNF, the ARC low‐order assemblies accumulate within dendritic spines but further from PSD‐95, suggesting a potential contribution to stabilization of spine actin rather than association with protein component of the post‐synaptic density. The low‐order assemblies align with those reported by Mergiya et al., 2023 [28] and might represent ARC monomers or very small oligomeric assemblies. Quantitative techniques, such as qPAINT [52] or RESI [53], would be necessary for accurate stoichiometric quantification.

While the role of ARC in AMPA receptor endocytosis is well established, its potential involvement as an oligomer has not been explored in neurons. Using the oligomerization‐incompetent ARC linker–CTD mutant, we show that ARC localizes to the post‐synaptic density predominantly as a low‐order assembly rather than as a monomer. Although this mutant preserves binding sites for synaptic interaction partners, it fails to accumulate at synapses and leads to an increased amount of AMPA receptors at the surface (Figure 4).

These findings suggest that ARC low‐order oligomerization contributes to its synaptic localization and is likely important for its role in AMPAR endocytosis. This agrees with recent models [54], which propose that ARC low‐order oligomers (tetramers) might be the minimal functional units required for AMPARs endocytosis, whereas higher‐order oligomers (32‐mers) may be associated with enhanced LTD. In our data, high‐order ARC assemblies and semi‐circular organization are preferentially observed in endocytic zones rather than in direct association with the PSD‐95 lattice, further supporting this distinction. In contrast, dimeric ARC might, instead, contribute to spine actin stabilization during LTP [54].

ARC semi‐circular assemblies at the EZ might represent ARC molecules involved in endocytic events [4, 67], but whether this involves a direct interaction with lipid membranes or the involvement of an obligate third molecular interaction partner was not clear. In fact, ARC is known to interact with proteins possessing a BAR domain—a structure known to sense and stabilize membrane curvature—such as Endophilin‐3 and PICK‐1 [7, 9, 47]. Here, we show with atomistic simulation and in vitro experiments with GUVs that ARC interacts with lipid bilayers – preferentially with PIP2‐rich membranes. While palmitoylation is not required for membrane localization, it enhances ARC recruitment to membranes, as shown in GPMVs and coarse‐grained simulation results. DTT‐derived GPMVs show only low‐order assemblies and no ARC membrane accumulations, suggesting that palmitoylation may promote membrane recruitment of ARC low‐order assemblies, potentially nucleating or stabilizing high‐order ones. We also observe that ARC induces both inward and outward tubulation in GUVs, consistent with the ability to remodel membranes, as further supported by molecular dynamic simulations (Figure 5). Since the GUV outer surface models, the inner leaflet of cellular membranes, this tubulation is consistent with a possible role in the endocytic processes. While Hedde et al. [46] observed only inward vesiculation with monomeric ARC, pointing at the budding out in extracellular vesicles release, our experiments using an oligomerization‐competent ARC show a predominance of outward tubulation—consistent with endocytic involvement. While these observations strongly support the ability of ARC to remodel membranes, they do not directly demonstrate that ARC induces functional endocytosis in neurons. Our data are consistent with a membrane‐deforming activity that could facilitate endocytosis, in line with the previous work by Eriksen et al. [16] showing that ARC oligomerization is required for transferrin endocytosis in heterologous cells. However, whether ARC alone is sufficient or necessary to drive cargo internalization in neurons remains to be established. Future studies, for example live‐cell imaging of AMPA receptor internalization in neurons expressing oligomerization‐deficient mutants, will be needed to test this possibility directly.

Finally, the observed 60–80 nm high‐order assemblies in neurons could be consistent with ARC viral‐like capsids of 60–120 units, observed so far only in vitro. This is in accordance with the oligomeric size of 130 molecules reported by Eriksen et al. [16] from in vitro quantifications, and partially with the 32‐mers shown by Zhang et al. [20]. Following studies involving quantitative techniques such as qPAINT or RESI will be necessary for accurate stoichiometric quantification of high‐order ARC assemblies.

Thus, different ARC oligomerization states likely serve distinct functions depending on synaptic context and activity state. Low‐order assemblies mediate association and AMPAR trafficking, while higher‐order oligomers are enriched in endocytic zones and may be linked to LTD‐related processes. In contrast, BDNF‐induced ARC predominantly accumulates within dendritic spines as low‐order assemblies, where it likely contributes to actin stabilization during LTP‐like states.

## 4 Methods

All experiments were performed in accordance with animal welfare guidelines set forth by Karolinska Institutet and were approved under the Ethical Permit nr 2645‐2021 by the Swedish board of agriculture (Jordbruksverket). Rats were housed with food and water available ad libitum in a 12‐hour light/dark environment.

### 4.1 Primary Mature Neuronal Culture

Primary cultures were prepared from embryonic day 18 (E18) Sprague Dawley rat embryos. The pregnant mothers were sacrificed with CO_2_ inhalation and aorta cut, and brains were extracted from the embryos. Cortexes or hippocampi were dissected and mechanically dissociated in Minimum Essential Medium, MEM (Thermo Fisher Scientific, 21090022). 2×10^5^ cells per 60 mm culture dish were seeded on poly‐D‐ornithine (Sigma Aldrich, P8638) coated #1.5 18 mm glass coverslips (Marienfeld, 0117580), and were let to attach in MEM with 10% horse serum (Thermo Fisher Scientific, 26050088), 2 mM L‐Glut (Thermo Fisher Scientific, 25030‐024) and 1 mM sodium pyruvate (Thermo Fisher Scientific, 11360‐070), at 37°C at an approximate humidity of 95%–98% with 5% CO_2_. After 2–4 h, coverslips were flipped over an astroglial feeder layer (grown in MEM supplemented with 10% horse serum, 0.6% glucose, and 1% penicillin‐streptomycin) and maintained in Neurobasal (Thermo Fisher Scientific, 21103‐049) supplemented with 2% B‐27 (Thermo Fisher Scientific, 17504‐044), 2 mM L‐glutamine and 1% penicillin–streptomycin. The neuronal cultures were treated with 5 µM 5‐fluorodeoxyuridine (FDU) at DIV 2–3, to prevent glia overgrowth. The cultures were kept for up to 24 days and fed twice a week by replacing one‐third of the medium per well: up to DIV7 with Neurobasal complete medium, and after DIV7 with BrainPhys (STEMCELL tech. 05790), 1% Pen/Strep (Gibco 15140‐114) and SM1 Supplement (STEMCELL tech. 05711). For experiments performed with immature cortical neurons (DIV 6–9), 100×10^3^ cells per well were seeded in 12 well plates on a poly‐D‐ornithine coated #1.5 18 mm glass coverslips. 3 hours post‐plating the media was changed to Neurobasal Medium supplemented with 2% B‐27 (Thermo Fisher Scientific, 17504‐044), 2 mM l‐Glutamine and 1% Penicillin‐Streptomycin (Sigma Aldrich, P4333).

### 4.2 Cell lines Culture

HeLa (ATCC CCL‐2) cells, kindly gifted by Önfelt Lab and tested mycoplasma fee, were cultured in DMEM (Thermo Fisher Scientific, no. 41966029) supplemented with 10% (vol/vol) fetal bovine serum (Thermo Fisher Scientific, no. 10270106), 1% penicillin/streptomycin (Sigma‐Aldrich, no. P4333) and maintained at 37°C and 5% CO_2_ in a humidified incubator. Cells were plated on #1.5 18 mm glass coverslips (Marienfeld, 0117580) 48h before imaging. HeLa cells were used because, as reported by Human Protein Atlas (www.proteinatlas.org/ENSG00000198576‐ARC/cell+line), they have null expression of the protein ARC compared to other cell lines.

### 4.3 Plasmid constructs

Plasmids used for ARC exogenous expression in neurons and HeLa cells are: pAAV‐EF1a_Arc‐FL‐C‐SNAP‐IRES‐WGA‐Cre, pAAV‐EF1a_Arc‐IM‐fusion‐SNAP‐IRES‐WGA‐Cre, pSFV‐SCA_Arc‐C‐AlfaTag, pAAV‐EF1a_Arc‐linker‐CTD‐SNAP‐IRES‐WGA‐Cre, pSFV‐SCA_5’‐UTR_Arc‐IM‐fusion‐SNAP_3’UTR, pSFV‐SCA_5’‐UTR_mEGFP‐Arc_3’UTR, and pGL4.11‐Arc7000‐mEGFP‐Arc‐UTRs (Kawashima et al., 2009, kindly provided by Prof. H. Bito from Department of Neurochemistry, Graduate School of Medicine, University of Tokyo) for neuronal expression was a gift from Michael Ratz (Karolinska Institute). Plasmids were prepared from the transformants and verified via Sanger sequencing. Details of the plasmids cloning are provided in Note S1.

### 4.4 Neuron Transfection and Live Cell Staining

DIV6‐9 neurons were transfected using Lipofectamine 2000 Transfection Reagent (Thermo Fisher Scientific, 11668019), according to the instructions of the manufacturer. For labelling fusion proteins, 24 hours after transfection, neurons were washed in Artificial CerebroSpinal Fluid (ACSF) and labelled with 5 µM of the SNAP substrates (New England BioLabs, SNAP‐Cell 647‐SiR), for 45 min at 37°C. Then, neurons were washed three times with ACSF and put back in the conditioned medium for at least 20 min. Mature primary cortical neurons were infected with a modified Semliki Forest Virus 16–18 hours before the experiment. Alternatively, neurons were transfected using calcium phosphate co‐precipitation protocol as reported [55]. In short: DNA (2 µg per coverslip) was diluted in TE solution (Tris‐HCl, pH 7.5, 10 mM; EDTA, pH 8.0, 1 mM). CaCl_2_ (2.5 M in 10 mM HEPES) was added to a final concentration of 250 mM. The mixed solution was added to 2× HEBS (HEPES Buffered Saline, pH 7.2). Neurons were pre‐incubated in 200 µl of conditioned medium from their culture dish with 50 µl of 5× Kynurenic acid stock (10 mM dissolved in unsupplemented culture medium) in a well of sterile MW12 and placed back in the incubator until the precipitate was ready. The precipitate was then added dropwise to the cells and incubated for 3–4 h. In order to stop the transfection, a 5:1 mix of BrainPhys without SM1 and Kynurenic acid was pre‐warmed. Then, 5 M HCl was added until the solution turned yellow. After removal of the transfection medium, the acidic medium was added to each coverslip which was further incubated at 37°C/5% CO_2_ for 15–20 min. After the incubation period the neurons were transferred back to the original Petri dish containing the conditioned medium and the construct was let express for 24–48 h at 37°C/5% CO_2_.

### 4.5 HeLa Transfections

For transfection, 2 × 10^5^ cells per well were seeded on coverslips in a six‐well plate. After one day cells were transfected using FuGENE (Promega, E2311) according to the manufacturer's instructions. 24–36 h after transfection cells were washed in phosphate‐buffered saline (PBS) solution, placed with phenol‐red free Leibovitz's L‐15 Medium (Thermo Fisher Scientific, 21083027) in a chamber and imaged.

### 4.6 GPMVs Production

To produce GPMVs, HeLa cells were plated in 60 mm petri dishes and transfected to express ARC‐C‐EGFP. Cells were washed twice with GPMVs buffer medium (150 mM NaCl, 10 mM Hepes, 2 mM CaCl). Cells in GPMVs buffer either adding 2 NEM (1M) to reach 2 mM final NEM concentration or 4% PFA and DTT (1M) to reach final concentration respectively of 25 mM for PFA and 2 mM for DTT. Cells were put back at 37°C at an approximate humidity of 95%–98% with 5% CO_2_ for at least 1h. GPMVs production visually assessed in a tabletop transmission light microscope. ARC‐C‐EGFP expressing HeLa cells‐derived NEM GPMVs further subdivided in two populations: homogeneous population, in which there were no resolvable ARC‐C‐EGFP clusters; and heterogenous population where instead, we could resolve ARC‐C‐EGFP clusters.

### 4.7 Rat ARC Protein Purification

The constructs psfARC‐c001 (pGEX‐6p1‐GST‐ArcFL, plasmid #119877 obtained from Addgene) or psfARC‐c008 (His‐Tev‐GST‐3C‐ArcCTD) were transformed into *E. coli* BL21 (DE3) T1R pRARE2 cells. The cells were cultivated in 3000 ml Terrific Broth (TB) medium supplemented with 8 g/l glycerol and Ampicillin (50 µg/ml), Chloramphenicol (34 µg/ml). Inoculation of overnight cultures from fresh transformants followed the cultures grown at 30°C, 175 RPM overnight in the presence of 0.4% glucose. After 24h the cultures were grown in the LEX system. At different times, the OD was measured for the cultures, and the temperature was set to 18°C at OD 2. The protein expression was induced at approximately OD 3 (IPTG, final concentration of 0.5 mM). Protein expression continued overnight before the cells were harvested by centrifugation (10 min at 4500 × g). The lysis buffer (100 mM HEPES, 500 mM NaCl, 10 mM imidazole, 10% glycerol, 0.5 mM TCEP, pH 8.0 (1.5 ml buffer per gram cell pellet) and the complete stock solution (1 ml per 1.5 l culture: 1 tablet Complete EDTA‐free (protease inhibitor cocktail, Roche) and 50 µl benzonase nuclease cell resuspension (PSF) per 1 ml) were added and the cell pellets were re‐suspended on a shaker table (cold room). The resuspended cell pellets were frozen at −80°C. The frozen cell pellets were briefly thawed in water (room temperature) and cells were disrupted by pulsed sonication (4s/4s, 4 min, 80% amplitude). The sonicated lysates were centrifuged (20 min at 49000 × g), and the soluble fractions were decanted and filtered through 0.45 µm filters. The clarified lysate was loaded onto a GSTrap 4B 5 ml column (1 ml/min, GE Healthcare) and the flow‐through was collected and re‐loaded at an additional time. The column was washed with GST wash buffer 1 (PBS, 0.5 mM TCEP, pH 7.4 and 2 (PBS, 1 M NaCl, 0.5 mM TCEP, pH 7.4, 4 ml/min). The column was then equilibrated with 10 column volumes of 3C protease cleavage buffer (50 mM Tris, 150 mM NaCl, 0.5 mM TCEP, 0.5 mM DTT, pH 8.0) (at 8°C), 3C protease (PSF, psfNTx3C‐c001, 1:500 molar ratio) was added and the column was incubated overnight at 8°C. The GSTrap‐column was then coupled to a 1 ml Histrap HP column (GE Healthcare), for the purpose of removing the 3C protease, and the target protein was eluted with 3C protease cleavage buffer (reverse GST purification). Finally, an elution step with GST‐elution (50 mM Tris, 150 mM NaCl, 30 mM reduced glutathione, pH 8.0 (at 8°C) buffer was performed, to check if some uncleaved protein remained bound to the column. Selected fractions were examined by SDS‐PAGE and fractions containing the cleaved target protein were pooled. The protein containing fractions from the reverse GSTrap purification were loaded onto the ÄKTA Xpress and purified overnight in gel filtration column HiLoad 16/60 Superdex 200 (GE Healthcare). Fractions containing the target proteins were pooled and concentrated with Vivaspin concentration filters (Vivascience 10 kDa cut off). The final concentration was measured (Nanodrop), the protein was flash frozen in aliquots of 100 or 200 µL in liquid nitrogen and stored at ‐80 °C. The final batch buffer was constituted by 50 mM Tris, 150 mM NaCl, pH 7.4 (at room temperature).

### 4.8 NHS Labelling

ARC purified proteins were thawed on ice and storage buffer was exchanged to PBS in Pierce Zeba Desalt Spin Columns (0.5 mL, ThermoFisher). Abberior STAR RED NHS was dissolved in DMSO at 10 mg/ml. 0.1 ml of 1M NaHCO3 (in water pH 8.5) solution was added for each 1 ml of protein (100 ug). 1 ul of the dye solution was added to the protein solution with NaHCO3 and incubated for 1 hour at room temperature with continuous stirring. Unbound dye was further removed via centrifugation in the desalting columns, and the final concentration assessed via Nanodrop.

### 4.9 GUV production

GUVs were produced as previously reported in Erdinc et al., 2015. Briefly, 1 mg mL^−1^ lipid solutions were prepared in chloroform (100% POPC, 95% POPC + 5% POPS, 95% POPC + 5% PIP2). Then, 5 µL of this solution were dried onto two parallel platinum wires mounted in a GUV Teflon chamber. A 300 mm sucrose solution was added to the chamber and a 10 Hz current was applied to the wires for an hour. GUV preparation was formed at room temperature. ARC ‐STAR635P (4 ug) was incubated with GUVs for 20 min before imaging.

### 4.10 Confocal Microscopy

#### 4.10.1 Confocal and FCS Setup

Confocal imaging and FCS measurement were performed at Confocal Microscope Zeiss LSM 780. Argon laser with 488 nm lines was used to excite EGFP samples and HeNe 633 nm laser was used to excite 647SiR or JF646.

### 4.11 STED microscopy

#### 4.11.1 Microscope Setup for STED

STED nanoscopy has been performed either at a Leica TCS SP8 3X STED, which is equipped with a HC PL APO 100x/1.40 Oil STED White objective, or at a custom‐built STED setup, based on a STED setup previously described [45]. *STED*: Excitation of the red‐shifted dyes was done with a pulsed diode laser at 640 nm with a pulse width of 60 ps (LDH‐D‐C‐640, PicoQuant, Berlin, Germany), excitation of the red dyes was done with a pulsed diode laser at 561 nm with a pulse width of 60 ps (PDL561 Abberior Instruments). Depletion was done with a pulsed 775 nm laser beam with a pulse width of 530 ps (KATANA 08 HP, OneFive GmbH, Regensdorf, Switzerland). Fast on and off control of the excitation and depletion lasers for STED is done using an AOTF (AOTFnC‐400.650‐TN + MPDS4C‐B66‐22‐74.156, AA Opto Electronic, Orsay, France) and AOM (MT110‐B50A1.5‐IR‐Hk + MDS1C‐B65‐34‐85.135‐RS, AA Opto Electronic, Orsay, France) respectively. The depletion beam is shaped using a vortex phase mask on a spatial light modulator (LCOM‐SLM X10468‐02, Hamamatsu Photonics, Hamamatsu, Japan). A λ/4 and a λ/2 wave plate are used to create the circular polarization necessary for optimal depletion focus formation. Fast galvanometer mirrors are used for scanning in STED imaging (galvanometer mirrors 6215H + servo driver 71215HHJ 671, Cambridge Technology, Bedford, MA, USA) in a scanning system allowing constant resolution across an 80×80 µm^2^ FOV as described previously [45]. The fluorescence is decoupled with a dichroic mirror into two channels. Channel 1 (magenta) has a notch filter (NF03‐785E‐25, Semrock), a bandpass filter (ET705/100 m, Chroma), and the fluorescence is focused onto a free space APD (SPCM‐AQRH‐13‐TR, Excelitas Technologies, Waltham, MA, USA). Channel 2 (green) has a notch filter (ZET785NF, Chroma), a bandpass filter (ET615/30 m, Chroma), and the fluorescence is focused onto a 62.5 *µm* core diameter multi‐mode fibre (M31L01, Thorlabs) coupled to an APD (SPCM‐AQRH‐14‐FC, PerkinElmer, Waltham, MA, USA). *General*: The setup uses a 100x/1.4 oil immersion objective (HC PL APO 100x/1.40 Oil STED White, 15506378, Leica Microsystems, Wetzlar, Germany) and a microscope stand (DMi8, Leica Microsystems). The system also uses a mechanical stage for moving of the sample in the lateral dimensions (SCAN IM 130×85–2 mm, Märzhäuser, Wetzlar, Germany) and a piezo to move the sample along the axial dimension (LT‐Z‐100, Piezoconcept, Lyon, France). The images were recorded by exciting AbberiorSTAR580 and 647SiR or AbberriorSTAR635P. Two‐color STED images were recorded line‐by‐line, adding up 4 lines with 0.03 msec pixel dwell time. The pixel size of the images is 19.53 nm.

#### 4.11.2 Neuron Sample Preparation for STED Imaging

For the live labelling of AMPA receptors, 1 µl of GluA antibody (1 mg/ml, GluA antibody – 182 411, Synaptic System) and 1 µl of FluoTag‐X2 anti‐mouse KLC (5 µM, cat. N1202) conjugated to Abberior STAR580 dye were pre‐incubated with 98 µl of pre‐conditioned neuronal medium. Neurons were, then, incubated with the AMPA labelling solution for 10–20 min in a humidified chamber at 37°C. After the incubation time neurons were left to recover for 5 min in their original medium and washed twice with artificial cerebrospinal fluid (ACSF) before the imaging or before the fixation. Before fixation, cells were washed once with 1X PBS, pre‐warmed at 37°C and fixed in pre‐warm 4% PFA at room temperature for 10 min. After being washed twice with PBS, cells were permeabilized by incubation with 0.5% TRITON X100 in PBS for 5 min or 0.1% TRITON X100 in PBS for 10 min. Blocking was performed by incubation with 5% bovine serum albumin in PBS (5% BSA/PBS) for 30 min at RT. Primary antibodies as Anti‐PSD‐95 antibody (Santa Cruz, sc‐32290, 1:50) and Anti‐ARC (Synaptic System, 156 003, 1:1000) was incubated in 5% BSA/PBS for 1 hour at RT. After three 5 min washes in PBS, the sample was incubated with donkey anti‐mouse Alexa594 secondary antibody (ThermoFisher A‐21203, 2 mg/ml, 1:200), goat anti‐rabbit Abberior STAR635P, or Anti‐GFP nanobody (FluoTag‐X4 anti‐GFP conjugated with Abberior STAR635P, N0304) and anti‐PSD‐95 nanobody (Nanotag, N3702‐Ab580‐L, 1:500) in 5% BSA/PBS for 1 hour at RT. The samples were washed in PBS for 15 min, and the coverslips were mounted I Mowiol.

#### 4.11.3 DNA‐PAINT

##### 4.11.3.1 Microscope Setup for DNA‐PAINT

Imaging was carried out using an inverted microscope (Nikon Instruments, Eclipse Ti2) equipped with a Perfect Focus System using the objective‐type TIRF configuration with an oil‐immersion objective (Nikon Instruments, Apo SR TIRF 100X, NA 1.49, oil). A 561 nm laser (MPB Communications, 1 W) was used for excitation and was coupled into a single‐mode fiber and into a Nikon manual TIRF module. The laser beam was passed through a cleanup filter (Chroma Technology, ZET561/10) and coupled into the microscope objective using a beam splitter (Chroma Technology, ZT561rdc). Fluorescence light was spectrally filtered with an emission filter (Chroma Technology, ET600/50m) and imaged with an sCMOS camera (Andor, Zyla 4.2 plus) without further magnification, resulting in an effective pixel size of 130 nm after 2×2 binning. The camera readout sensitivity was set to 16‐bit and the readout bandwidth to 200 MHz. The central 1024×1024 pixels (512×512 after binning) of the camera were used as the region of interest. 3D imaging was performed using an astigmatism lens (Nikon Instruments, N‐STORM) in the detection path [56]. Image acquisition and microscope control was performed using µManager [57] (Version 2.0.1). The illumination angle was set into a highly inclined illumination (HILO) mode.

##### 4.11.3.2 Neuron Sample Preparation and Labeling Scheme for DNA‐PAINT

After fixation, neurons were quenched using 100 mM NH_4_Cl (Merck, 12125‐02‐9) in PBS. Then, samples were washed three times with PBS and incubated with 0.1% Triton X‐100 in PBS for 20 min for permeabilization. Afterward, the samples were washed with PBS, and 3% BSA in PBS, supplemented with 0.05 mg/ml sheared salmon sperm DNA was applied for blocking. For drift‐correction gold nanoparticles (1:3 dilution in PBS) were incubated for 5 min. Primary antibodies (ClathrinHC) were incubated with secondary nanobodies (Rabbit IgG Nanotag) and direct nanobodies (GFP 1H1 and GFP 1B2 and PSD‐95 from Nanotag) in 300 µl antibody incubation buffer overnight onto the sample. Following the overnight incubation, the sample was washed 4 times with PBS and once with Buffer C+.

##### 4.11.3.3 Buffers

The following buffers were used for sample preparation and imaging:

- Buffer C+: 1× PBS, 500 mM NaCl and 0.05% Tween‐20, 1x PCA, 1x PCD, 1x Trolox
- Buffer C: 1× PBS, 500 mM NaCl
- Antibody Incubation buffer: 1×PBS, 1 mM EDTA, 0.02% Tween‐20, 0.05% NaN_3_, 2% BSA and 0.05 mg/ml sheared salmon sperm DNA

##### 4.11.3.4 PCA, PCD and Trolox

Trolox (100×) was made by the addition of 100 mg of Trolox to 430 µl of 100% methanol and 345 µl of 1 M NaOH in 3.2 ml of water. PCA (40×) was made by mixing 154 mg of PCA in 10 ml of water and NaOH and adjustment of pH to 9.0. PCD (100×) was made by the addition of 9.3 mg of PCD to 13.3 ml of buffer (100 mM Tris‐HCl pH 8.0, 50 mM KCl, 1 mM EDTA, 50% glycerol).

##### 4.11.3.5 Nanobody‐DNA Conjugation Via Single Cysteine

Nanobodies against GFP (cat: N0305), tagFP (cat: N0501), rabbit and mouse IgG (cat: N2405 & N2005) were purchased from NanoTtag Biotechnologies with a single ectopic cysteine at the C‐terminus for site‐specific and quantitative conjugation. The conjugation to DNA‐PAINT docking sites was performed as described previously [58]. First, buffer was exchanged to 1× PBS + 5 mM EDTA, pH 7.0 using Amicon centrifugal filters (10k MWCO) and free cysteines were reacted with 20‐fold molar excess of bifunctional maleimide‐DBCO linker (Sigma Aldrich, cat: 760668) for 2–3 hours on ice. Unreacted linker was removed by buffer exchange to PBS using Amicon centrifugal filters. Azide‐functionalized DNA was added with 3–5 molar excess to the DBCO‐nanobody and reacted overnight at 4°C. Unconjugated nanobody and free azide‐DNA was removed by anion exchange using an ÄKTA Pure liquid chromatography system equipped with a Resource Q 1 ml column. Nanobody‐DNA concentration was adjusted to 5 µM (in 1xPBS, 50% glycerol, 0.05% NaN3) and stored at ‐20°C.

#### 4.11.4 Neuron Imaging for DNA‐PAINT

Imaging was performed in sequential rounds of DNA‐PAINT with the parameters summarized in Table S2.

### 4.12 STARSS

#### 4.12.1 Microscope Setup for STARSS

The STARSS experiments [26] were performed in a custom confocal microscopy system equipped with a dual channel polarized detection (parallel and perpendicular polarizations).

#### 4.12.2 STARSS Measurements

The anisotropy decay in the μ‐second time domain was measured using rigid rsEGFP2 fusion of the ARC protein. At every scanning position a polarized pulse scheme of light was delivered to the sample: at the beginning of the sequence the fluorescent protein was fully switched off using 1.5 millisecond of 488 nanometer light at roughly 20 kW/cm^2^, then roughly 10% of the protein was switched on using a short burst of 250 nanosecond of linearly polarized 405 nm light at roughly 110 kW/cm^2^ (parallel direction), finally fluorescence was induced using 1.5 millisecond of circularly polarized 488 nanometer light at roughly 20 kW/cm^2^. The fluorescence photons were recorded with a binning time of 1 microsecond.

### 4.13 RNA‐FISH

#### 4.13.1 Probe Design and Production

Arc RNA FISH probes were designed based on rat transcriptome as previously reported by Gelali [59], and they are reported in Supplementary table S1.

#### 4.13.2 iFISH

RNA FISH was performed as reported in Gelali et al., 2019 [59]. Briefly, neurons were fixed fix in 4% PFA/0.4xPBS (‐Ca, ‐Mg), for 10 min at RT and then incubate in 1xPBS/0.5% Triton X‐100 / RVC for 20 min. Cells were further incubated in in the RNA WASH 25% buffer for 5 min followed by primary hybridization mix (1 ul of primary probe, 25 uM, for 100 ul o RNA hyb buffer) for 24 h at 30 C. Wash in RNA WASH 25% buffer followed for 30 min at 30C. The same was performed for the secondary hybridization mix (1 ul of secondary probe conjugated to Alexa 594 dye, 2 uM, for 100 ul o RNA hyb buffer) with an additional 60 min incubation with RNA WASH 25% buffer and a rinse with 2xSSC buffer. A standard immunofluorescence staining as described above followed by label Arc‐EGFP. Hybridization buffer (RNA HYB 25%): 10% Dextran sulfate (Sigma #D8906), 25% Formamide (Ambion #AM9342), 2x SSC (Ambion # AM9765), 1 mg/ml E.coli tRNA (Sigma #R4251), 0.02% BSA (Ambion #AM2616), 2 mM Ribonucleoside vanadyl complex (NEB S1402S), up to 10 ml Nuclease‐free water (Ambion #AM9932). Wash buffer (RNA WASH 25%)(pH:7‐8): 25% Formamide (Ambion #AM9342), 2x SSC (Ambion # AM9765), 32.5 ml Nuclease‐free water (Ambion #AM9932).

#### 4.13.3 Molecular Modeling

The prediction of the structure of ARC NTD was determined using the Robetta webserver. We employed atomistic and coarse‐grained molecular dynamics simulations as well as the HADDOCK docking method. The 24–134 aa of Uniprot sequence Q63053 was predicted by the TrRosetta [49] method available through (https://robetta.bakerlab.org) at the confidence level of 0.71. The two helices of both structures are well aligned with RMSD of 3.36 Å (CE alignment) or 2.72 Å (SALIGN), as computed in PyMod plugin in PyMol.

Both atomistic and coarse‐grained molecular dynamics simulations were employed and carried out using Gromacs 2020.3 (atomistic simulations) or 2019.4 (coarse‐grained) [60]. The atomistic simulations considered the NTD, either non‐palmitoylated or containing palmitoyl groups at residues C94, C96, and C98, in proximity to a symmetric lipid bilayer with area 10×10 nm^2^ mimicking the composition of the intracellular plasma membrane leaflet (5% DOPC, 5% POPC, 20% DOPE, 20% POPE, 7% DOPS, 8% POPS, 10% POPI24, 25% cholesterol) [61] bathed in a 150 mM KCl solution. All systems were built with the charmm‐gui web server using the Charmm36m force‐field [62]. The simulations were run in the NPT ensemble following the charmm‐gui default molecular dynamics parameters for the equilibration and production steps. The production step was 400 ns long with the first 100 ns already excluded from the reported simulation. The coarse‐grained simulations were based on the MARTINI force‐field version 2.6 that includes the parameters for palmitoylated cysteines [63]. Three systems were considered. In the first, 4 triple‐palmitoylated ARC NTDs were positioned at the interface of a 40 × 40 nm^2^ membrane bathed in 150 mM NaCl (see Figure 6A). The membrane consisted of an asymmetric distribution of ∼60 different types of lipids, including PIP lipids, and mimicked the realistic composition of an average mammalian plasma membrane [64]. In the second and third system, respectively, 4 non‐palmitoylated or 4 palmitoylated ARC NTDs were positioned in solution next to a 30 × 30 nm^2^ membrane devoid of PIP lipids (see Figure 5D). Following equilibration steps these systems were simulated for 5–10 µs in two replicas. All systems were prepared and simulated following protocols from http://cgmartini.nl/ [65, 66]. No restraints were imposed on the protein secondary structure. The simulated trajectories were visualized in VMD [67] and analyzed using custom scripts based on MDAnalysis [68].

Arc‐FL, ARC truncated mutants and multimers structures were predicted taking advantage of Colab notebook (https://colab.research.google.com/github/deepmind/alphafold/blob/main/notebooks/AlphaFold.ipynb), it uses a slightly simplified version of AlphaFold v2.1.0 [50]. Structures and prediction errors were then visualized using ChimeraX (v1.4rc202205290614).

### 4.14 Quantification and Statistical Analysis

#### 4.14.1 Confocal Image Analysis: AMPA Puncta Intensity Analysis

Single plane images or stacks of confocal images (separated by 250 nm) were taken from fixed samples expressing ARC‐FL, ARC‐linker‐CTD‐SNAP, lifeact‐YFP, and live stained for AMPA receptors. 30×30 um manually cropped ARC or lifeact images were binarized upon thresholding using the Haung algorithm to identify neurites. Binary masks were then enlarged by 0.25 µm and only AMPA signals within that area were taken into account. AMPA puncta were identified using Maxima plugin of ImageJ (prominence: 2). The photon‐count of the local Maxima defines the AMPA intensity. For Arc‐FL and Arc‐linker‐CTD, each data point represents the average AMPA puncta intensity per neuron divided by ARC intensity (N_linker‐CTD_ = 29, N_Arc‐FL_ = 22, from 2 independent primary cortical cultures).

#### 4.14.2 FCS Data Analysis

FCS correlation curves obtained from ARC‐C‐EGFP‐expressing HeLa cells‐derived GPMVs were analysed using the software FoCuS_point (version 1.13.156). The data were fitted with a mono exponential diffusion equation (A1 fixed to 1 and AR1 fixed to 6). Diffusion coefficient (D) was then derived from the diffusion time (τ_D_) using the formula D = ω^2^/(8*lg2* τ_D_), where ω is the confocal spot diameter measured for each experimental session. Triplets were excluded moving the residual tau threshold to 10^−2^ msec.

#### 4.14.3 ARC Assemblies Size Retrieving Model From Diffusion Coefficients

As reported by Galiani al., 2022 [69] the transit diffusion time for a cytosolic 3x monomeric EGFP (80.85 kdal) in GPMVs is 39 ± 13.5 µm^2^/ s. We applied the Stokes‐Einstein equation adapted for the masses as reported by Young et al., 1980 as it follows:

$$M_{2}=M_{1}\;{\left(\frac{D_{t1}}{D_{t2}}\right)}^{3}$$

where M_1_ and M_2_ are the respective species’ masses, while *D*_*t*1_ and *D*_*t*2_ are their measured diffusion coefficients. From that we estimated first the diffusion coefficient of ARC‐C‐EGFP (72.4 kdal) monomer as being 40.5 µm^2^/ s and then the ARC assemblies for each GPMVs conditions.

#### 4.14.4 STED Image Analysis: ARC Nanoclusters Size, Intensity, and Marker Colocalization

The analysis of ARC nanoclusters size and intensity for immature and mature primary neurons for the different ARC labelling strategies was performed using the Fiji distribution of ImageJ and using the script provided, see Code Availability. Each nanocluster was localized using the Find Maxima plugin of ImageJ (prominence: 10). The photon‐count of the local Maxima defines the intensity of the clusters. For each nanocluster, a 2D Gaussian equation was fitted in a 5 by 5 pixels patch centered on the Maxima coordinates, in x and y. FWHM in x and y were averaged to get the mean FWHM reported in the text. The data was then processed using Matlab (using the script provided) and Microsoft Excel. For the colocalization of ARC nanoclusters with another marker (Arc mRNA, AMPA receptors or PSD‐95), the STED images in the channel of the marker were analysed using the script provided. A Laplatian filtering (smoothing: 3) and a Gaussian Blurring (sigma: 1) were first applied to the images, each image was then auto‐thresholded and binarized. In binary images, the Watershed and morphological (radius: 1) filters were applied and just particles larger than 0.01 um were detected and enlarged by 0.05 um. The areas identified are the areas in which local ARC maxima are found in ARC channel images. ARC nanoclusters within defined areas (colocalization with Arc) and outside (extrasynaptic ARC nanoclusters) are separately analysed using the same script as reported above in order to identify the size of ARC nanoclusters which are or not co‐localizing with the other marker. For the identification of EZ, PSD‐95 areas were radially enlarged of 200 nm, each PSD‐95 area was then subtracted from its enlarged counterpart, ARC local maxima found in the resulting area were attributed to the EZ. The percentage of ARC nanoclusters in colocalization with Arc mRNA is calculated dividing the numbers of clusters in colocalization over the total number of nanoclusters detected in STED imaging.

The line profiles were traced using a smart neighborhood of three pixels with the software Imspector (v0.1) and fitted with a GaussAmp function using OriginLab software.

#### 4.14.5 DNA‐PAINT Image Analysis

Raw DNA‐PAINT data was reconstructed to super‐resolution images with the Picasso software package (version up to 0.8.0) [70], latest version available at https://github.com/jungmannlab/picasso). Drift correction was performed with a redundant cross‐correlation following gold particles as fiducials for cellular experiments. Alignment of sequential imaging rounds was performed using gold particles.

ARC, Clathrin and PSD‐95 clusters of localizations were obtained using the DBSCAN algorithm [71]. DBSCAN parameters, minimum localizations, and clustering radius were selected based on the imaging parameters of the individual super‐resolution channel of the protein. Background regions were taken as a base for determining the cluster minimum localization number and both DBSCAN parameters were further adjusted based on visual validation to determine a cutoff value distinguishing background from specific protein clusters. Cluster volumes are calculated by finding a 3D convex hull and extracting its volume. Distances between clusters of different protein types were calculated subsequently. Alternatively, the SMLM clusterer algorithm present in Picasso software was used to measure 1st NNDs between ARC and PSD‐95 single molecules. Default parameters such as minimum number of localizations (10), cluster radii of 10 nm (xy) and 25 nm (z) were used.

Quantification of ARC accumulation at the synaptic level was obtained from DNA‐PAINT images measuring the local density of ARC single molecules (counted with ImageJ local maxima finder) in colocalization with PSD‐95 areas in comparison to extra synaptic ARC densities. Just the synapses where ARC single molecules were 30% more abundant than in extrasynaptic areas were considered synapses with ARC accumulations. ARC molecules to PSD‐95 nanoclusters alignment was quantified upon tracing 120 nm averaged line profiles along PSD from axially sliced and 65 nm y‐projected images of top‐viewed synapses.

#### 4.14.6 STARSS Data Analysis and Statistics

A mono‐exponential fitting function was used to model the data,

$$r\;\left(t\right)=r_{0}\;expexp\;\left(-\frac{t}{\tau_{R}}\right)+r_{\infty },$$

where *r* is the fluorescence anisotropy computed from the signals recorded in the parallel and perpendicular channels, *t* is time, τ_*R*_ is the rotational diffusion time, *r*_0_ is the anisotropy that decays with time constant τ_*R*_ in the observed time window, and *r*_∞_ is the anisotropy fraction that does not decay in the observed time window.

Signals from 56 fields of views of 20×20 micrometer squared, recorded from 28 neurons derived from 3 independent cultures in four sessions in different days, were manually segmented in spines and dendrites. The signals were spatially integrated and summed together to obtain time decays representative of the full dataset. Errors are computed propagating the photon shot noise of the detectors in the anisotropy formula [26]. All the FOV with total counts less than 3×10^4 were discarded. For the discarded FOV the confidence interval computed by propagating the photon shot noise was already high, and the background subtraction was challenging and inducing unwanted biases. This estimation of the structure size of the fraction of anchored protein is obtained by converting the rotational diffusion time to the hydrodynamic diameter using the Stokes‐Einstein equation [26], assuming room temperature and viscosity of water. The fitting parameters are reported in the following table.

**Table:** 

Segmentation	τ_*R*_ (us)	*d_R_* (nm)	*r*_0_	*r*_∞_
Dendrites	29.6	61	0.011	0.0069
Spines	88.6	88	0.010	0.0070

For statistical significance of anisotropy decays in dendritic spines and shaft we applied the modified Chi‐squared method reported by Hristova et al., 2023 [72] to measure differences between arbitrary curves for the scenario of a single measurement at each point with known standard deviation.

### 4.15 Statistical Analysis

The statistical tests applied to compare pooled data from multiple replicas were the two‐sample two‐sided Student's t‐test for normally distributed data and the non‐parametric Kolmogorov–Smirnov test for non‐normally distributed data [73]

## Author contributions

I.T. conceived research and supervised the project. M.D. cultured and labeled neurons, optimized the labeling protocols, performed the cloning and the imaging experiments. G.C. cultured the neurons, produced the viruses and optimized the labelling and immunostaining protocols. M.S. cultured the neurons, the glial cells, optimized the labeling protocols and the Calcium‐Phosphate transfections protocol. J.A. built the STED optical set‐up and performed the ARC nanoclusters density analysis. C.S. performed the imaging experiments with EVs markers together with M.D. E.S. produced and performed the GUVs, GPMVs and FCS experiments together with M.D. L.R performed the molecular dynamics simulations supervised by L.D. L.A.M, E.M.U. and R.K. performed DNA‐PAINT experiments and data analysis together with M.D. supervised by R.J, A.V. performed the STARSS measurement together with M.D. M.D. analyzed the data and wrote the manuscript together with I.T. and with the input from all the authors.

## Conflicts of Interest

The authors declare no conflict of interest.

## Resource Availability–Lead Contact

Further information and requests for resources and reagents should be directed to and will be fulfilled by the lead contact, Ilaria Testa (ilaria.testa@scilifelab.se).

## Material Availability

Plasmids generated in this study will be deposited to Addgene and reported with the name and catalog number or unique identifier.

## Data and Code Availability

● Microscopy data reported in this paper will be shared by the lead contact upon request.

● All original code is available in this paper's supplemental information.

● Any additional information required to reanalyze the data reported in this paper is available from the lead contact upon request.

## Supporting information




**Supporting File**: advs75187‐sup‐0001‐SuppMat.docx

## Data Availability

The data that support the findings of this study are available from the corresponding author upon reasonable request.
