# Supplementary material for: Nanoscale Spatial Organization of ARC High‐ and Low‐Order Assemblies at Excitatory Synapses
Source: Adv Sci (Weinh). 2026 Apr 7:e20740. Online ahead of print. doi: 10.1002/advs.202520740 (PMC13334648; doi:10.1002/advs.202520740)
Supplement: Supplementary file 1 — Supporting File: advs75187‐sup‐0001‐SuppMat.docx [file ADVS-9999-e20740-s001.docx]

**Supporting Information**

**Nanoscale Spatial Organization of ARC High- and Low-Order Assemblies at Excitatory Synapses**

*Martina Damenti^1^, Giovanna Coceano^1^, Mariline Mendes Silva^1^, Jonatan Alvelid^1^, Chiara Sgattoni^1^, Andrea Volpato^1^, Lea Rems^2^, Luciano A. Masullo^3^, Eduard M. Unterauer^3,4^, Rafal Kowalewski^3,4^, Lucie Delemotte^1^, Erdinc Sezgin^5^, Ralf Jungmann^3,4^, Ilaria Testa ^1^ **

M.D., G.C., M.M.S., J.A., C.S., A.V., L.D., I.T.

^1^Department of Applied Physics and Science for Life Laboratory, KTH Royal Institute of Technology, 100 44 Stockholm, Sweden

L.R.

^2^Faculty of Electrical Engineering, University of Ljubljana, 1000 Ljubljana, Slovenia

L.A.M., E.U., R.K., R.J.

^3^Max Planck Institute of Biochemistry, 82152 Planegg, Germany

E.U., R.K., R.J.

^4^Faculty of Physics and Center for NanoScience, Ludwig Maximilian University, Geschwister-Scholl-Platz, D-80539 München, Germany

E.S.

^5^Department of Women's and Children's Health, Karolinska Institutet, 171 77, Stockholm, Sweden

* Corresponding author: Ilaria Testa

**List of content**

**Supplementary notes**

Supplementary Note 1. Cloning design and constructs

Supplementary Note 2. Confocal image analysis of synaptic GluA puncta in colocalization with PSD-95

Supplementary Note 3. BDNF stimulation of primary cortical neurons.

**List of Figures**

Figure S1. Organization and different expression levels of ARC in primary neuronal cultures.

Figure S2. ARC is organized in nanoclusters in primary neuronal cultures.

Figure S3.1 ARC nanoscale organization at the synaptic compartment at basal activity level and upon BDNF stimulation.

Figure S3.2 ARC nanoscale organization at the molecular scale with 3D DNA-PAINT.

Figure S4.1. GluA accumulates in punta-like clusters at synaptic sites in colocalization with PSD-95 density sites.

Figure S4.2 ARC induces membrane inward bending and ARC linker-CTD expression affects AMPA receptors surface levels.

Figure S5.1 Palmitoylation and PIP lipids modulate ARC-membrane interaction.

Figure S5.2 Palmitoylation and PIP lipids are not required to mediate ARC-membrane interaction.

Figure S5.3 ARC induces tubulations in GUVs.

**List of Tables**

Table S1. ARC mRNA FISH probes

Table S2. DNA-PAINT imaging parameters

**Supplementary notes**

**Supplementary Note 1. Cloning design and constructs**

- pAAV-EF1a_ARC-FL-C-SNAP-IRES-WGA-Cre was generated by first exchanging mCherry from p83_pAAV-EF1a-mCherry-IRES-WGA-Cre (kindly provided by Michael Ratz from Department of Cell and Molecular Biology, Karolinska Institutet) with SNAP amplified from the plasmid template Sec61β-SNAP (kindly provided by Francesca Bottanelli, [Freie Universität Berlin](https://scholar.google.com/citations?view_op=view_org&hl=en&org=3273064866052637217)) using the primer pair

5′- atgactggatccATGGACAAAGACTGCGAAATGAAGC

5’- agtcatgaattcCTAACCCAGCCCAGGCTTGCCC

Both backbone and insert were digested using BamHI-HF and EcoRI-HF (NEB) separately, followed by T4 ligation (NEB) and transformation of NEB Stable cells (NEB). Then, rat ARC was amplified from pcDNA3.1(+)-C-eGFP-ARC using the primer pair.

5’- atgactggtaccggattggccaccATGGAGCTGGACCATATGACG

5’- agtcatggatccgctaccgctgccTTCAGGCTGGGTCCTGTCACT

Both backbone and insert were digested using BamHI-HF and KpnI-HF (NEB) separately, followed by T4 ligation (NEB) and transformation of NEB Stable cells (NEB).

- pAAV-EF1a_ARC-IM-fusion-SNAP-IRES-WGA-Cre was generated digesting the backbone pAAV-EF1a_ARC-FL-C-SNAP-IRES-WGA-Cre using NcoI (NEB) and amplifying three sequences from it using the following three pairs of primers:

ARC_CTD_rev_ g: 5’- tatcgataagcttgatatcgttaTTCAGGCTGGGTCCTGTC

ARC_NTD_fwd_g: 5’- tcaggtgtcgtgaggtaccgggtttagtgaaccgtGCCACCATGGAGCTGGACC

ARC_NTD_rev_SNAP: 5’- ttcatttcgcagtctttgtcgccgccgatggagccGGACTCCAGGCGGTCG

ARC_SNAP_fwd: 5’- gggccgaccgcctggagtccggctccatcggcggcGACAAAGACTGCGAAATGAAGCGCAC

ARC_SNAP_rev: 5’- actgggtacttgccgcccatgatggagccgccACCCAGCCCAGGCTTGCC

ARC_CTD_fwd_SNAP: 5’- tgggcaagcctgggctgggtggcggctccatcATGGGCGGCAAGTACCCA

Backbone and PCR products were assembled with Gibson Assembly (Master Mix, NEB).

- pAAV-EF1a_ARC-linker-CTD-SNAP-IRES-WGA-Cre was generated from pAAV-EF1a_ARC-FL-C-SNAP-IRES-WGA-Cre digesting the backbone with NcoI (NEB) followed by T4 ligation (NEB) and transformation of NEB Stable cells (NEB).

pAAV-EF1a_ARC-linker-CTD-SNAP-IRES-WGA-Cre was generated from pAAV-EF1a_ARC-FL-C-SNAP-IRES-WGA-Cre digesting the backbone with NcoI (NEB) and BamHI-HF (NEB) and amplifying from it a sequence using the following primers pair:

5’- tgaggtaccggattggccacCCATGGAGCTGGACCATATGAC

5’- tcatttcgcagtctttgtcgggatccgctaccgctgccCCATGGACTCCAGGCGGT

The backbone and PCR product were assembled with Gibson Assembly (Master Mix, NEB) and and transformed with NEB Stable cells (NEB).

Both backbone and insert were digested using BamHI-HF and EcoRI-HF (NEB) separately, followed by T4 ligation (NEB) and transformation of NEB Stable cells (NEB). Then, rat ARC was amplified from pcDNA3.1(+)-C-eGFP-ARC using the primer pair.

5’- atgactggtaccggattggccaccATGGAGCTGGACCATATGACG

5’- agtcatggatccgctaccgctgccTTCAGGCTGGGTCCTGTCACT

Both backbone and insert were digested using BamHI-HF and KpnI-HF (NEB) separately, followed by T4 ligation (NEB) and transformation of NEB Stable cells (NEB).

- pAAV-EF1a_ARC-IM-fusion-rsEGFP2-IRES-WGA-Cre was generated digesting the backbone pAAV-EF1a_ARC-FL-C-rsEGFP2-IRES-WGA-Cre using NcoI (NEB) and amplifying three sequences from it using the following three pairs of primers:

ARC_CTD_rev_ g: 5’- tatcgataagcttgatatcgttaTTCAGGCTGGGTCCTGTC

ARC_NTD_fwd_g: 5’- tcaggtgtcgtgaggtaccgggtttagtgaaccgtGCCACCATGGAGCTGGACC

ARC_NTD_rev_rs2: 5’- agctcctcgcccttgctcac gccgccgatggagccgGACTCCAGGCGGTCGGCC

ARC_rsEGFP2_fwd: 5’- gggccgaccgcctggagtccggctccatcggcggcGTGAGCAAGGGCGAGGAG

ARC_rsEGFP2_rev: 5’- actgggtacttgccgcccatgatggagccgccCTTGTACAGCTCGTCCATGC

ARC_CTD_fwd_rs2: 5’- gcatggacgagctgtacaagggcggctccatcATGGGCGGCAAGTACCCA

The backbone and PCR products were assembled with Gibson Assembly (Master Mix, NEB).

- pSFV-SCA_5’-UTR_ARC-IM-fusion-SNAP_3’UTR was generated from Semliki-Forest Virus pSCA3-LifeAct-DronpaM159T (kindly gifted by Dr. Stefan W. Hell, MPI-BCP Göttingen, Germany) which was digested with XmaI-HF and NotI-HF (NEB). ARC-IM-fusion-SNAP sequence was amplified from pAAV-EF1a_ARC-IM-fusion-SNAP-IRES-WGA-Cre using the following primers pair:

5’- cggcgagcagATGGAGCTGGACCATATGAC

5’- gctggcccctTTATTCAGGCTGGGTCCTG

rat ARC-UTR and 3’-rat ARC-UTR sequences were amplified from costumed gene fragment cDNA purchased from IDT (Integrated DNA Technologies) using the two following couples of primers:

5’- cacagaattctgattggatcAGTGCTCTGGCGAGTAGTCC

5’- ccagctccatCTGCTCGCCGGGGTTACG

5’- gcctgaataaAGGGGCCAGCCCAGGGTC

5’-aattcaattaattaccctgcTTTAAATTTCATAGTTTTATTAACAAAATCATATATATGTATATATATATATATGTCTGTCTTTGAGGTAAGATGGTGTGGGCCAGATGG

The backbone and PCR products were assembled with Gibson Assembly (Master Mix, NEB).

- pSFV-SCA_ARC-FL-C-AlfaTag was generated from Semliki-Forest Virus pSCA3-LifeAct-DronpaM159T (kindly gifted by Dr. Stefan W. Hell, MPI-BCP Göttingen, Germany) which was digested with XmaI-HF and NotI-HF (NEB). ARC sequence was amplified from pAAV-EF1a_ARC-FL-C-SNAP-IRES-WGA-Cre using the following primers pair:

5’- cacagaattctgattggatcGCCACCATGGAGCTGGAC

5’- GGggatccgctaccgctgccttcaggctgggtcctgtcac

AlfaTag sequence is made by the annealing of two primers. Backbone and products were assembled with Gibson Assembly (Master Mix, NEB).

**Supplementary Note 2. Confocal image analysis of synaptic GluA puncta in colocalization with PSD-95**

Single plane confocal images were taken from fixed samples immunostained for GluA and PSD-95 and analyzed with Fiji/ImageJ. Single-channel PSD-95 images (135x135 μm) were first smoothed using a Gaussian Blur filter (sigma: 1). PSD-95 puncta were then detected using the Maxima plugin (prominence: 3). Detected maxima were used to generate binary maps, which were subsequently dilated to 5x5 pixels per maximum. These binary maps were used to identify individual PSD-95 densities using the Particle Analysis plugin. To identify extra-PSD-95 site, four additional binary maps were generated by shifting the original binary map byt 5 pixels in each of the four cardinal directions relative to the center. GluA intesity was measured form GluA single-channel images within each of the five binary maps, allowing quantification of GluA signal within PD95 densities (avarage GluA intensity within each puncta defined by the original binary map) and in sorrounding extra-synaptic regions (maximum GluA intensity detected within the four shifted binary maps).

**Supplementary Note 3. BDNF stimulation of primary cortical neurons.**

DIV22 primary cortical neurons were treated in their conditioned medium with 100 ng/ml of BDNF (STEMCELLS Biotechnology, Lot #1000038506 Catalog # Cytokines Human Recombinant BDNF Brain-derived neurotrophic factor 78005, in 0.1% BSA and milliQ water) or control solution (0.1% BSA in milliQ water) for 4h prior to sample fixation (4% PFA) followed by immunostaining.

**Supplementary Figures**


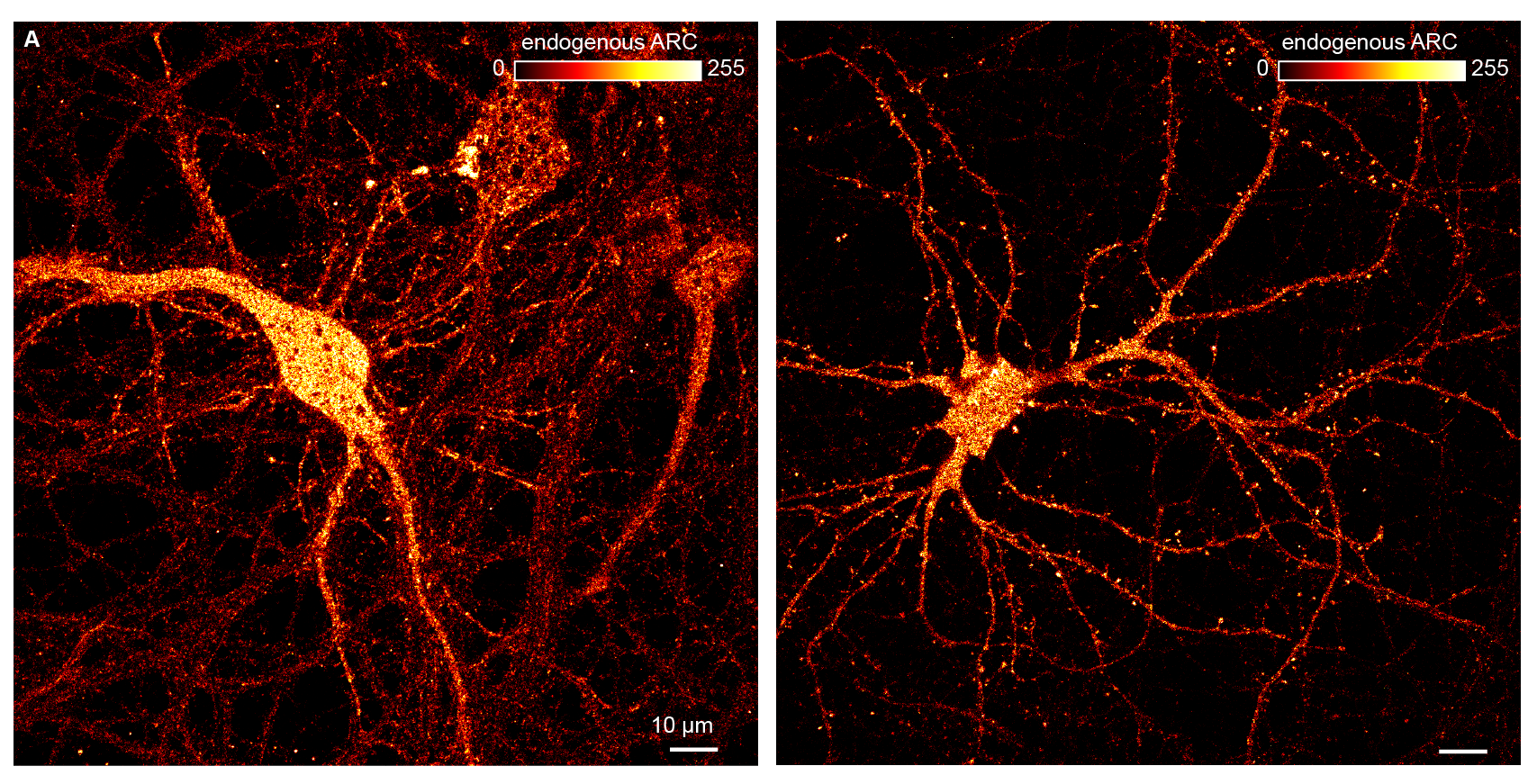


**Figure S1. Organization and different expression level of ARC in primary neuronal cultures.**

(A) Representative examples of primary cortical neurons (DIV22) where the protein ARC is immunostained and imaged in confocal microscopy. Variability in ARC expression levels from neuron to neuron is observed, together with its nuclear localization.


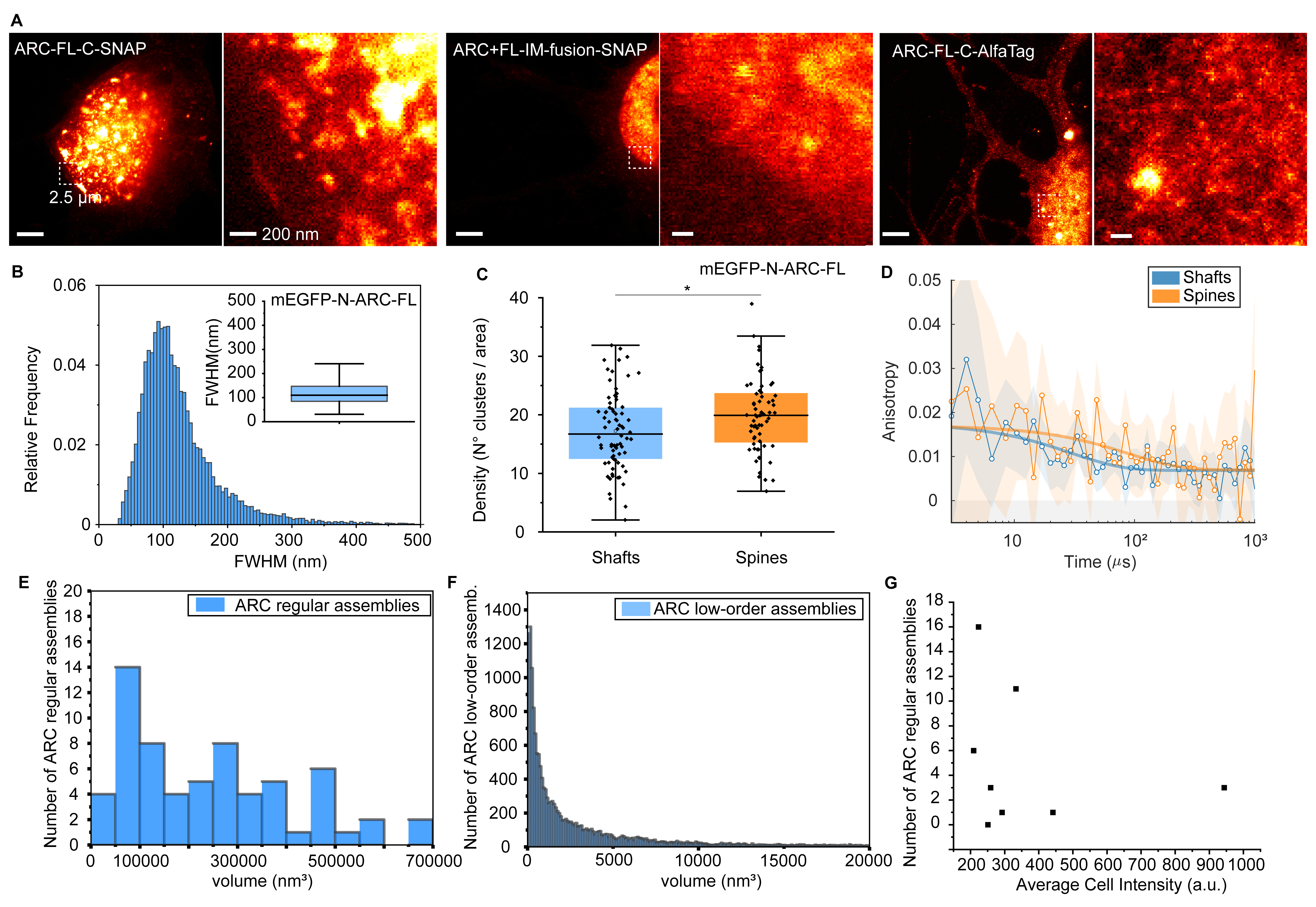
**Figure S2. ARC is organized in nanoclusters in primary neuronal cultures.**

1. Immature cortical neurons (DIV7-10) showing ARC localization in the nucleus for the different ARC labeling strategies: ARC-FL-C-SNAP (left panels), ARC-FL-IM-fusion-SNAP (central panels), ARC-FL-C-AlfaTag (right panels).
2. The histogram reports mEGFP-N-ARC-FL nanocluster FWHM distribution obtained from 23 neurons (DIV17-21) derived from 5 samples in 2 independent cultures.
3. The box plot represents the significantly higher density of mEGFP-N-ARC-FL nanoclusters in spines versus dendritic shaft. Each data plot is the average density value per spines and shaft per image. Two-sample two-sided Student’s t test p-value: 0.01708, the box plots show the 25–75% interquartile range, with the middle line representing the mean, and the whiskers derived from 1.5 * interquartile range t test p-value).
4. Anisotropy decays measured with STARSS and mono-exponentially fitted for ARC-FL-IM-fusion-rsEGFP2 in spines (orange) and in shafts (blue).
5. The histogram reports the volume of ARC regular structures (high-order oligomers) distribution as measured in 3D DNA-PAINT from 21 cortical neurons (DIV21-22) derived from 2 independent cultures.
6. The histogram reports the volume of ARC low-order oligomers distribution as measured in 3D DNA-PAINT from 21 cortical neurons (DIV21-22) derived from 2 independent cultures.
7. The scatter plot reports the lack of correlation between the number of detected regular structures and the overall mEGFP-N-ARC-FL cell expression level.


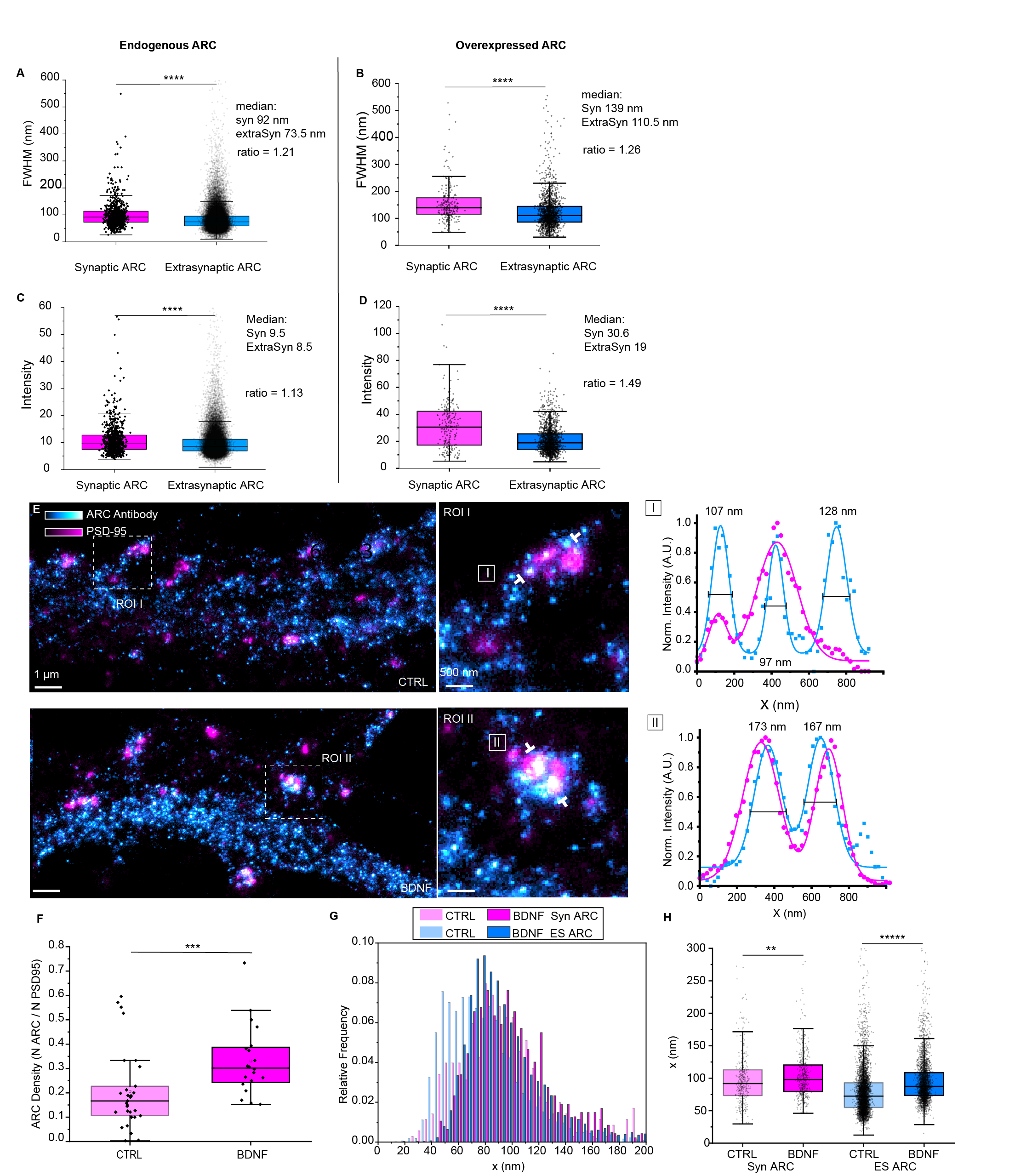


**Figure S3.1 ARC nanoscale organization at the synaptic compartment at basal activity level and upon BDNF stimulation.**

1. The box plot shows the significant difference in size between endogenous ARC nanoclusters co-localizing with PSD-95 (Synaptic ARC) and extrasynaptic ARC derived from 26 neurons derived from 3 independent cultures. The box plots show the 25–75% interquartile range, with the middle line representing the mean, and the whiskers derived from 1.5 * interquartile range. Two-sample two-sided Kolmogorov–Smirnov test p-value: 1.77e-41. It is also reported the ratio between the size of synaptic and extra-synaptic ARC nanoclusters, synaptic nanoclusters are 20% larger than extra -synaptic ones.
2. The box plot shows the significant difference in size between exogenous mEGFP-ARC nanoclusters co-localizing with PSD-95 (Synaptic ARC) and extrasynaptic ARC derived from the neuron shown in Fig. 2B. The box plots show the 25–75% interquartile range, with the middle line representing the mean, and the whiskers derived from 1.5 * interquartile range. Two-sample two-sided Kolmogorov–Smirnov test p-value: 1.06e-15. It is also reported that the ratio between the size of synaptic and extra-synaptic ARC nanoclusters, synaptic nanoclusters are 20% larger than extra-synaptic ones similarly to the endogenous counterpart.
3. The box plot shows the significant difference in intensity between endogenous ARC nanoclusters co-localizing with PSD-95 (Synaptic ARC) and extrasynaptic ARC derived from 26 neurons derived from 3 independent cultures. The box plots show the 25–75% interquartile range, with the middle line representing the mean, and the whiskers derived from 1.5 * interquartile range. Two-sample two-sided Kolmogorov–Smirnov test p-value: 1.69e-09. It is also reported the ratio between the intensity of synaptic and extra-synaptic ARC nanoclusters, synaptic nanoclusters are 13% brighter than extra -synaptic ones.
4. The box plot shows the significant difference in intensity between exogenous mEGFP-ARC nanoclusters co-localizing with PSD-95 (Synaptic ARC) and extrasynaptic ARC derived from the neuron shown in Fig. 2B. The box plots show the 25–75% interquartile range, with the middle line representing the mean, and the whiskers derived from 1.5 * interquartile range. Two-sample two-sided Kolmogorov–Smirnov test p-value: 1.10e-25. It is also reported the ratio between the intensity of synaptic and extra-synaptic ARC nanoclusters, synaptic nanoclusters are 49% brighter than extra-synaptic ones, the synaptic component is 36% brighter than the endogenous counterpart.
5. (top row) representative stretch of a primary cortical neuron (DIV22) where the organization of ARC (cyan) in nanoclusters is shown in relation to the synaptic marker PSD-95 (magenta). In ROI I, ARC nanoclusters of different sizes (107, 97 and 128 nm FWHM) are localized in close proximity to PSD-95. The line profile is traced along the white arrows. (bottom row) representative stretch of a primary cortical neuron (DIV22) where the organization of ARC (cyan) in nanoclusters is shown in relation to the synaptic marker PSD-95 (magenta) upon stimulation with BDNF (4h, 100 ng/ml). In ROI II, ARC nanoclusters of different sizes (173 and 167 nm FWHM) localize in close proximity to PSD-95. The line profile is traced along the white arrows.
6. The box plot reports that the density of ARC nanoclusters in close proximity to PSD-95 is significantly higher upon BDNF stimulation (magenta) compared to the control condition (pink). Each data point represents the average number of ARC nanoclusters per PSD-95 puncta per image (N_CTRL_= 31, from 11 neurons, N_BDNF_= 20, from 2 independent samples). Box plots show the 25–75% interquartile range, with the middle line representing the mean, and the whiskers derived from 1.5 * interquartile range. Two-sample two-sided Kolmogorov–Smirnov test p-value: 3.24x10^-4^.
7. The histogram reports the FWHM distributions of synaptic (Syn) and extra-synaptic (ES) ARC nanoclusters in control condition (CTRL, pink and light blue) and upon chronic stimulation with BDNF (4h, 100 ng/ml) (magenta and blue) (20 DIV22 primary cortical neurons from 2 independent samples).
8. The box plot shows that both synaptic (Syn, pink) and extra-synaptic (ES, blue) ARC nanoclusters exhibit a significant increase in size upon BDNF stimulation compared to the control condition. Box plots show the 25–75% interquartile range, with the middle line representing the mean, and the whiskers derived from 1.5 * interquartile range. Two-sample two-sided Kolmogorov–Smirnov tests, Syn CTRL vs BDNF p-values: 0.0072, ES CTRL vs BDNF p-values: 1.33 x10^-14^).


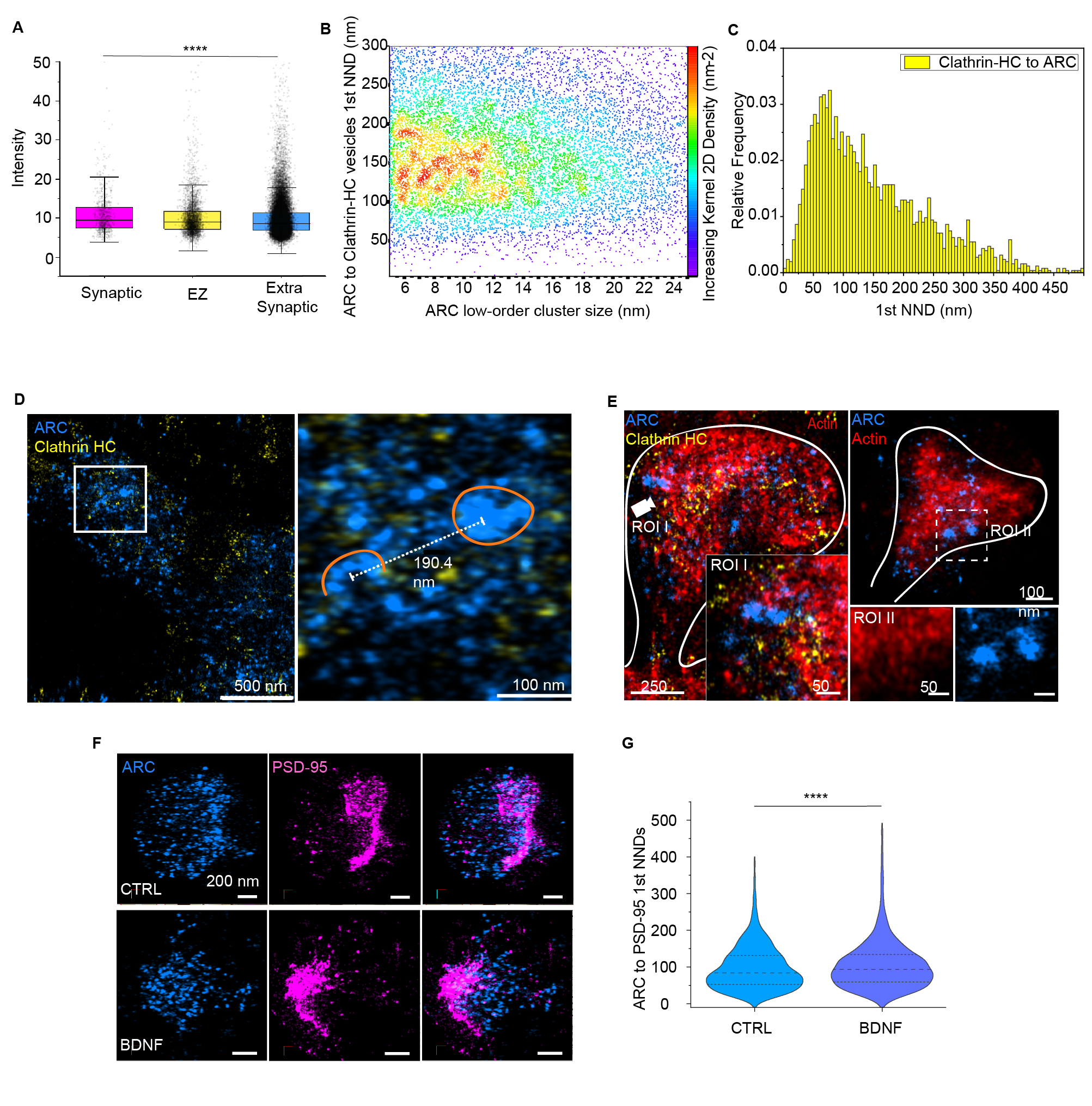


**Figure S3.2 ARC nanoscale organization at the molecular scale with 3D DNA-PAINT.**

1. The box plot of immunolabelled endogenous ARC shows significant difference in brightness between Synaptic ARC nanoclusters (co-localizing with PSD-95), ARC within the EZ (within 200 nm from PSD-95 perimeter) and extrasynaptic ARC from 26 neurons derived from 3 independent cultures. The box plots show the 25–75% interquartile range, with the middle line representing the mean, and the whiskers derived from 1.5 * interquartile range. Two-sample two-sided Kolmogorov–Smirnov test p-values respectively: p = 5.6487e-10, p = 2.1644e-04, p = 1.2136e-06).
2. The scatter plot shows the relationship between ARC to clathrin-coated vesicles 1^st^ NNDs to ARC, the density color map for each datapoint reveals several higher density areas corresponding to different ARC low-order cluster sizes located within 100 nm to 200 nm from clathrin-coated vesicles.
3. The histogram reports the distributions of 1^st^ NNDs from clathrin-coated vesicles to ARC which peaks at 75 nm.
4. Multiplexed 3D DNA-PAINT imaging of mEGFP-N-ARC-FL in primary cortical neurons. Representative example of ARC nanoscale arrangement in semi-circular organization together with ARC particle-like assemblies. In cyan: ARC, in yellow: Clathrin-HC. Clathrin HC is not extensively observed in proximity to ARC semi-circular assembly.
5. Multiplexed 3D DNA-PAINT imaging of mEGFP-N-ARC (cyan) and Clathrin-HC (yellow), together with peptide-PAINT of actin (red) in primary cortical neurons. Exemplar dendritic spines where ARC high-order assemblies are not seen in colocalization with Clathrin-HC and actin (left panel) or just with actin (right panel).
6. Multiplexed 3D DNA-PAINT imaging of mEGFP-N-ARC (cyan) and PSD-95 (magenta), in primary cortical neurons. (Top panels) Exemplar dendritic spines where ARC low-order assemblies are mostly seen in colocalization with PSD-95 in CTRL condition. (Bottom panels) Exemplar dendritic spines where ARC low-order assemblies are more spread all over the dendritic spine further away from PSD-95.
7. The violin plot reports the distribution of 1st NNDs from ARC low-order assemblies to PSD-95 single molecules. Upon BDNF stimulation (4h, 100 ng/ul, NBDNF = 29 spines), the distribution is significantly shifted towards larger values compared to a control condition (NCTRL= 27 spines). Fine dashed lines show the 25–75% interquartile range, with the middle line representing the median (medianBDNF:93.56 nm, median CTRL: 84.02 nm). Two-sample two-sided Kolmogorov–Smirnov test p-values: 8.23×10-4).


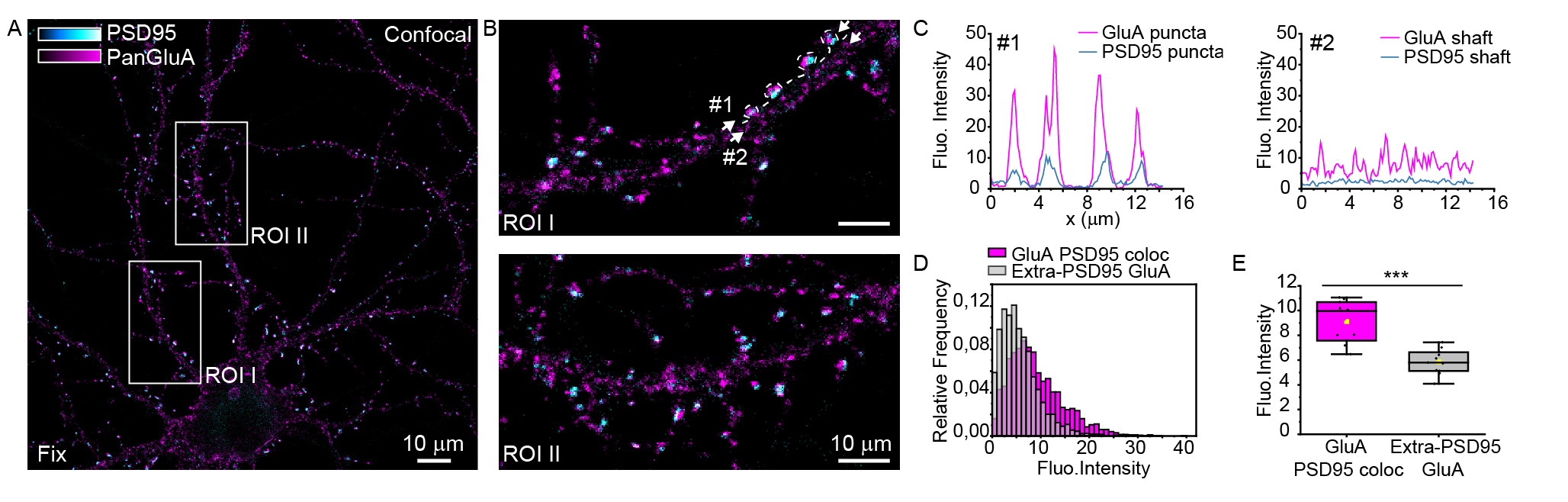
**Figure S4.1. GluA accumulates in punta-like clusters at synaptic sites in colocalization with PSD-95 density sites. (**A) Representative confocal image of primary cortical neurons (DIV20-21) immunostained for GluA subunits (PanGluA, magenta) and PSD-95 density sites (cyan).

(B) Two representative ROIs of dendritic filaments showing PSD-95 and GluA clusters at spines heads; spine morphologies are highlighted with a white line.

(C) Line profiles at the indicated signs in B (ROI I), comparing GluA intensity and clasterization at the level of spine heads (PSD-95 peaks) or in the dendritic shaft.

(D) The histogram reports the relative frequency of GluA cluster intensity in colocalization with PSD-95 (magenta) or outside PSD-95 density sites (gray). N= 2671 GluA clusters from 11 neurons.

(E) The box plot shows the significantly higher fluorescent intensity of GluA clusters, averaged per cell, in colocalization with PSD-95 (magenta), with respect to GluA outside the PSD-95 density site (gray). N= 11 neurons.


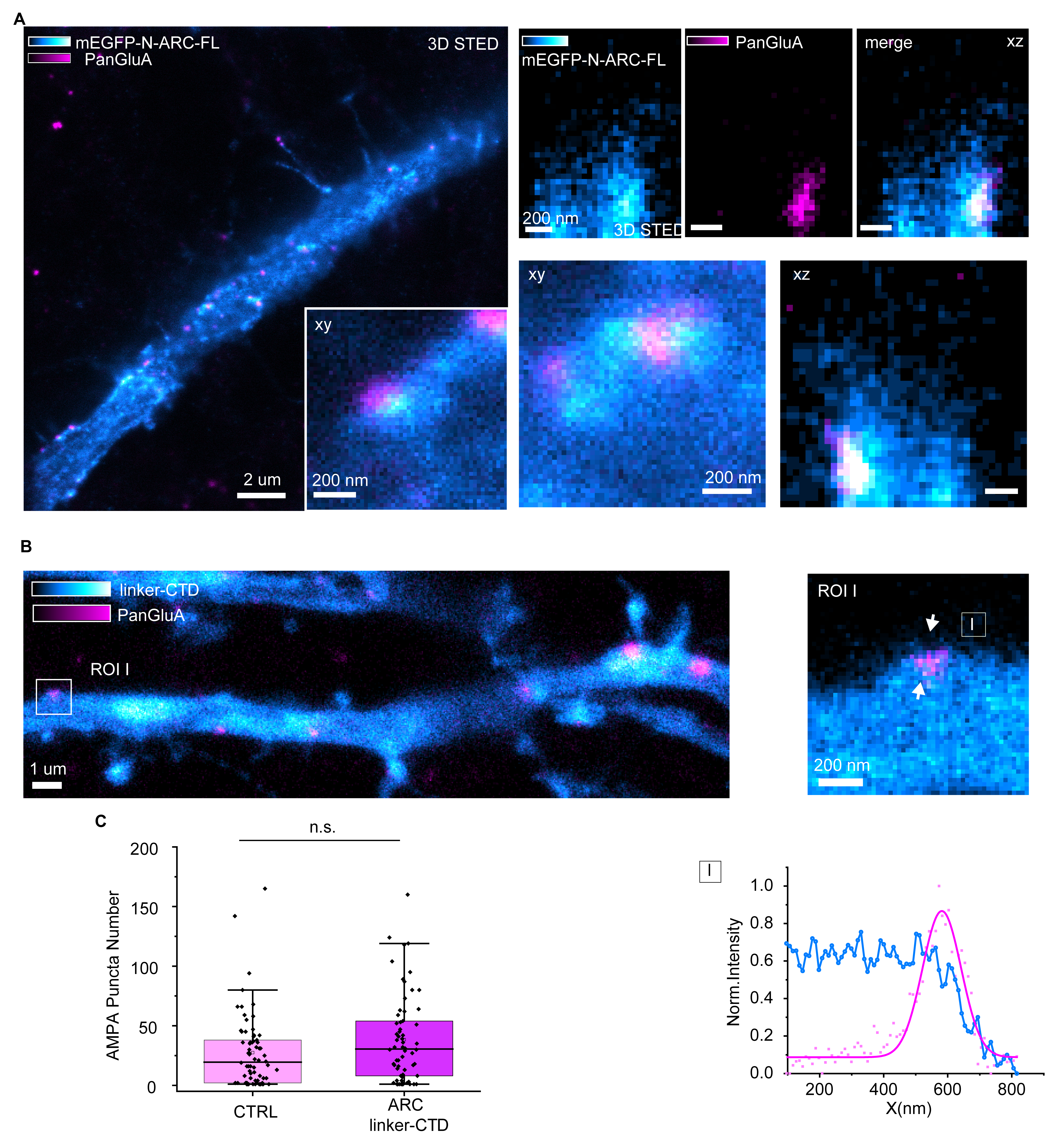


**Figure S4.2 ARC induces membrane inward bending and ARC linker-CTD affects AMPA receptors surface levels.**

1. Mature hippocampal neurite (DIV21) expressing mEGFP-N-ARC-FL (cyan) and PanGluA (magenta), live stained prior to fixation and imaged in 3D STED microscopy. ARC nanoclusters colocalization with PanGluA persists in 3D STED showing the independence from the geometry of the compartment.
2. Mature cortical neurite (DIV20-21) expressing ARC linker-CTD-SNAP (cyan) and PanGluA (magenta), live stained prior to fixation and imaged in STED microscopy. In ROI I, no ARC nanoclusters are seen in co-localization with GluA, line profile traced along the white arrows.
3. The box plot shows that puncta number in ARC linker-CTD-SNAP expressing neurons are not significantly different from the number in Lifeact-YFP expressing neurons (KS test p-value 7.4021e-31). Each data point represents the average AMPA puncta intensity per cell. 6 samples (N=55 neurons) from 2 independent primary cortical cultures.


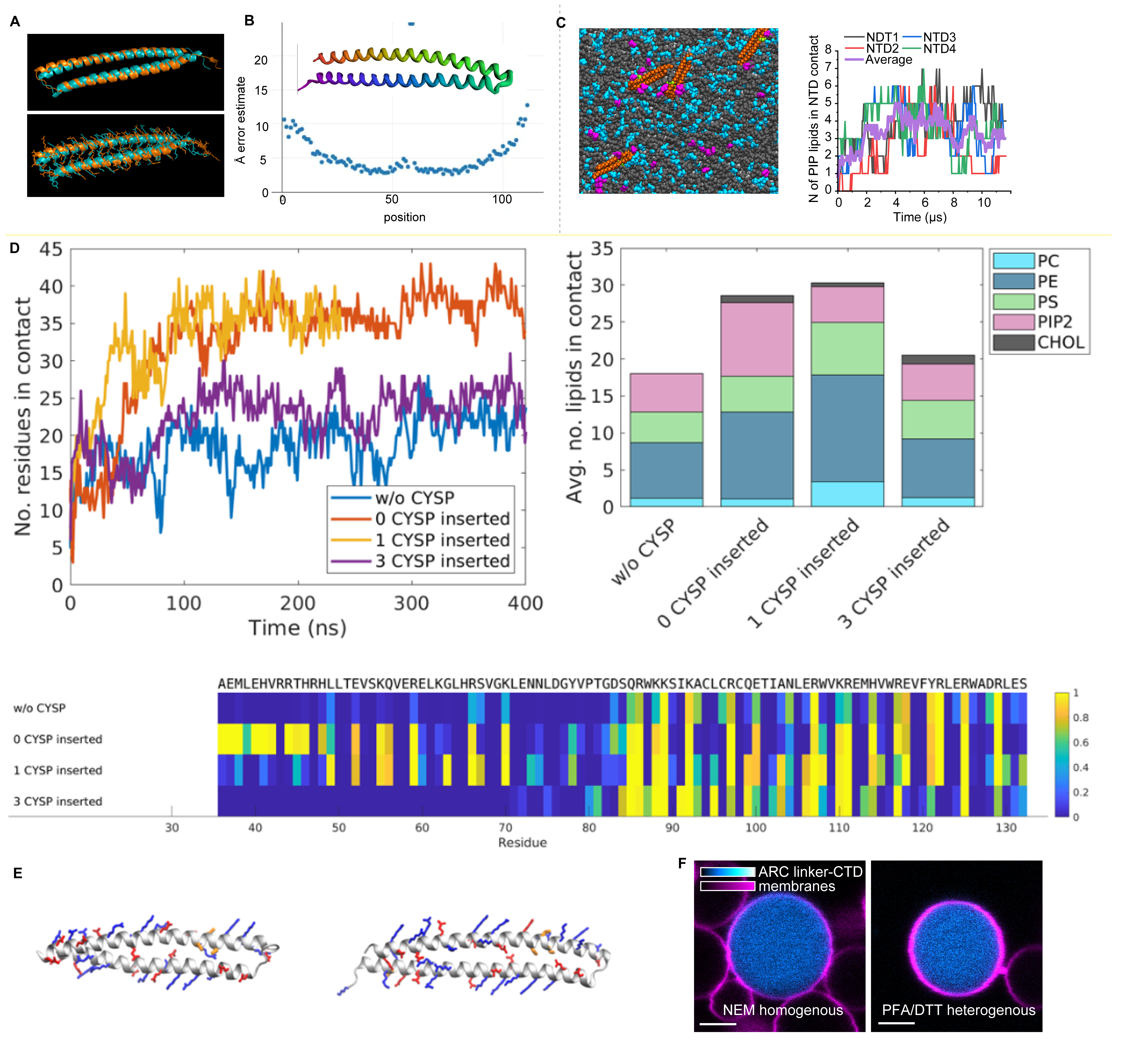


**Figure S5.1. Palmitoylation and PIP lipids modulate ARC-membrane interaction.**

1. Comparison between the NTD of rat ARC from AlphaFold (orange) and TrRosetta (teal). The top and bottom images show the same structures, but the bottom image omits the individual residues from the representation. The two helices of both structures are well aligned with RMSD of 3.36 Å (CE alignment) or 2.72 Å (SALIGN), as computed in PyMod plugin in PyMol.
2. Output from TrRosetta modeling of the ARC NTD. The input sequence consisted of amino acids 24-134 of the ARC rat sequence, the output structure yielded two alpha helices between amino acids 26 and 130.
3. (left panel) Increased density of PIP lipids (magenta) in proximity of NTDs (orange) at time = 11.8 us from the beginning of the simulation. Note that PIP lipids do not bind permanently to the NTDs but are able to detach and diffuse further along the membrane. (right panel) The number of PIP lipids bond vs. time for each of the 4 NTDs (thin colored lines) and the average over all NTDs (thick violet line).
4. Atomistic simulations with the ab initio ARC NTD model. Top left panel: The number of NTD residues that are bound to lipids vs. time, depending on the number of palmitoyl tails (CYSP) pre-inserted into the bilayer. Top right panel: The percentage of time spent by each NTD residue in contact with the membrane at the time interval 100-400 ns. Each pixel corresponds to a different residue. Each line of pixels corresponds to a different simulation (see the text on the left side). Residues colored in yellow tones spend most of the time in contact with the membrane. Bottom panel: The average number of lipids that are in contact with any residue of the NTD at the time interval 100-400 ns.
5. Models of ARC NTD predicted using the ab-initio method (left) and the TrRosettamethod (right) within the Robetta server. The negatively and positively charged residues are shown as red and blue sticks, respectively. The cysteine residues that become palmitoylated are shown as orange sticks.
6. Examples of NEM (left) or PFA/DTT (right)-produced GPMVs derived from ARC linker-CTD expressing HeLa cells, showing homogeneous ARC layout and no ARC micrometric clusters.


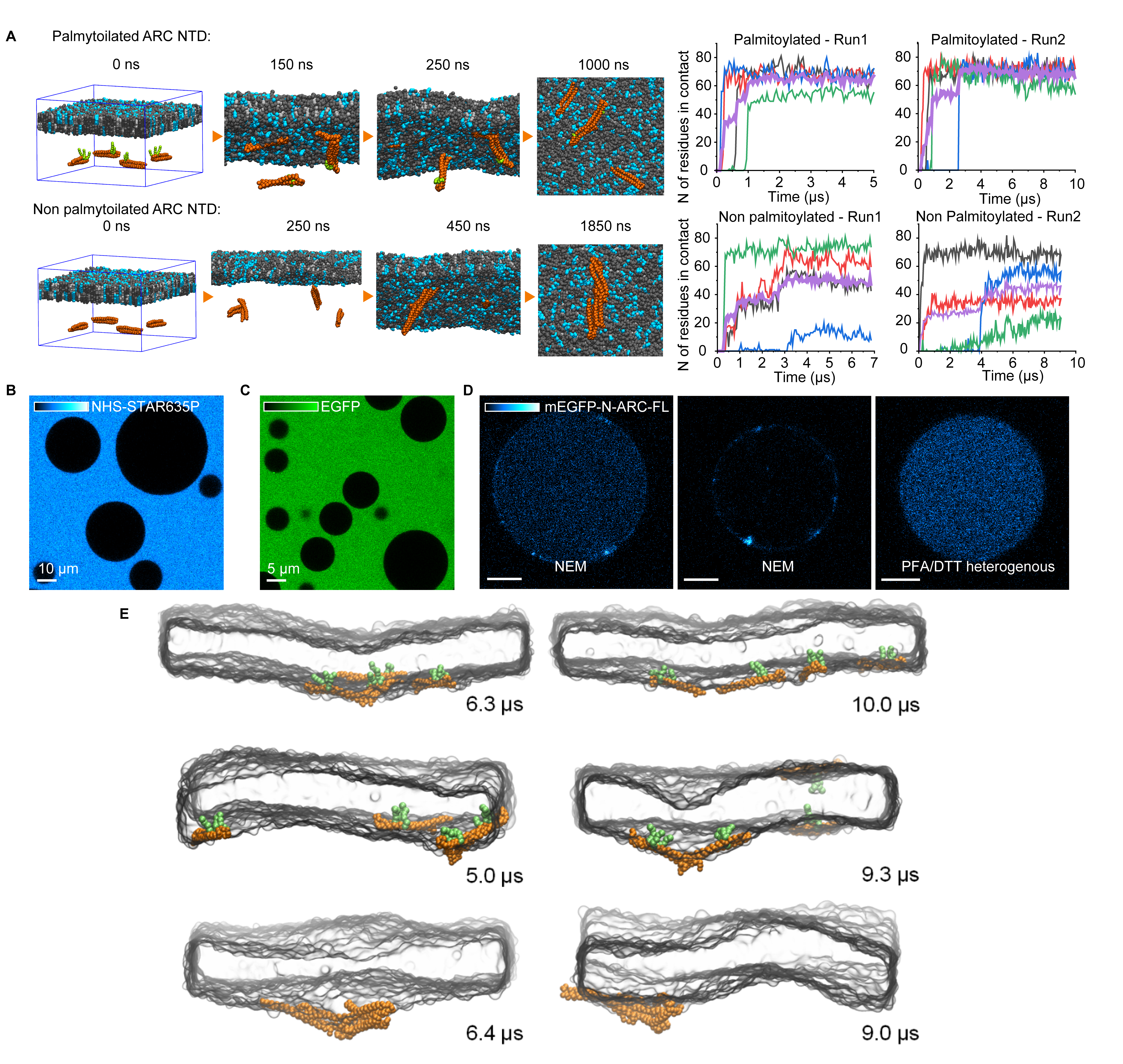


**Figure S5.2. Palmitoylation and PIP lipids are not required to mediate ARC-membrane interaction.**

1. Two coarse-grained molecular dynamics systems: (upper panel) palmitoylated NTDs and (lower panel) non-palmitoylated NTDs. In the palmitoylated NTDs simulation, two NTDs interact in solution with each other and one with the membrane at 150 ns. At 250 ns two of the NTDs are bound to the membrane. At 1000 ns, all four NTDs are bound to the membrane. In the non-palmitoylated NTDs simulation two of the NTDs interact in the solution at 250 ns. At 450 ns, the third NTD interacts with the first two, whereas the fourth binds to the membrane. The three interacting NTDs attach to the membrane at 1850 ns. The graphs on the right quantify the number of NTD residues in contact with the lipids for each of the NTDs and their average (thick violet line). The result was confirmed by two sets of simulations.
2. GUVs incubated with NHS-STAR635P showing absence of the interaction between the dye and the GUVs lipid bilayers.
3. GUVs incubated with EGFP showing absence of the interaction between the protein and the GUVs lipid bilayers.
4. (left and central panel) Examples of NEM-produced GPMVs derived from mEGFP-N-ARC-FL expressing primary cortical neurons (DIV21) showing comparable results to HeLa cells derived GPMVs: ARC micrometric clusters in proximity and co-localizing with the GPMVs membranes. (right panel) Example of PFA/DTT-produced GPMVs derived from mEGFP-N-ARC-FL expressing primary cortical neurons (DIV21) showing comparable results to HeLa cells derived GPMVs: no homogeneous ARC layout and no resolved ARC micrometric clusters.
5. Snapshots from coarse grained simulations. (top panels) System with palmitoylated ARC NTD interacting with a membrane containing PIP lipids. (middle panels) System with palmitoylated ARC NTD interacting with a membrane without PIP lipids, resulting from two independent simulations. (bottom panels) System with non-palmitoylated ARC NTD interacting with a membrane without PIP lipids, results from two independent simulations. The time within a simulation, at which each snapshot was taken, is indicated below each image.


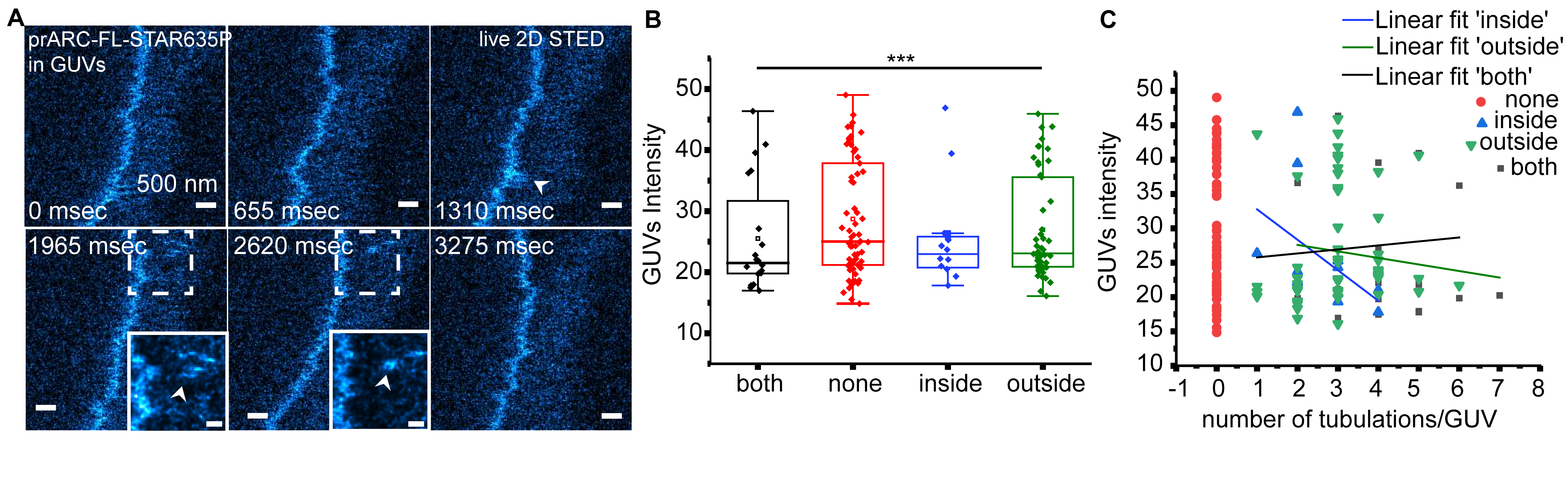


**Figure S5.3 ARC induces tubulations in GUVs**

1. STED time-lapse imaging of GUVs incubated with rat prARC-FL-STAR635P showing ARC dynamic interactions with lipid bilayers and budding out of vesicular organizations.
2. Box plot showing ARC-FL-STAR635P signal intensity at the GUV level for GUVs respectively inward (blue), outward (green) tubulations, both (black) or no tubulations (red) percentages. A non-significant difference in intensity is reported among the distinct tubulations groups.
3. Scatter plot reporting the non-significant correlations between ARC intensity at the GUV level and the number of observable tubulations imaged in confocal.


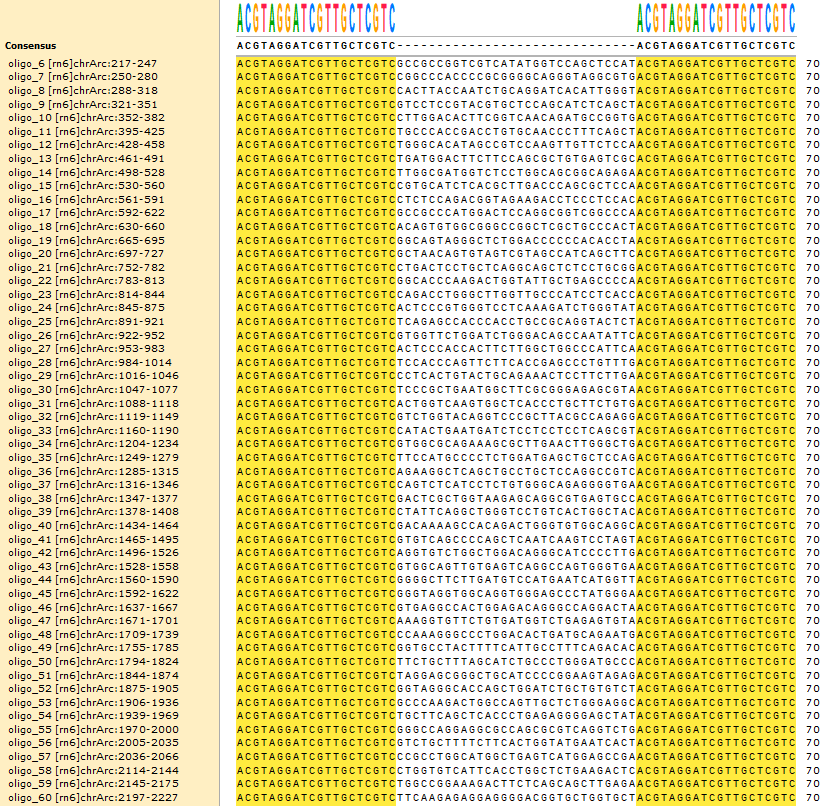


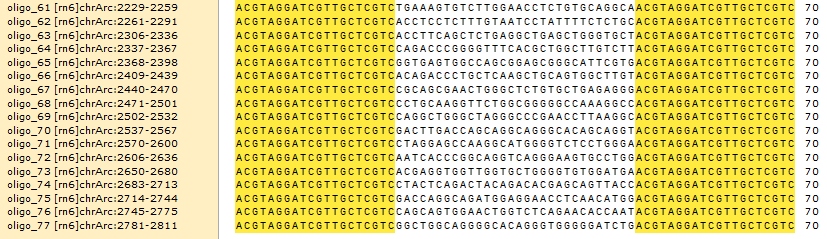


**Table S1. ARC mRNA FISH probe**

The table reports the RNA probes used in mRNA FISH experiment

| **Imaging session** | **Marker** | **Concentration** | **Power at BFP** | **Number of frames** |
| --- | --- | --- | --- | --- |
| Dataset_1_day1 | mEGFP-ARC | 35 pM (R3) | 15 mW | 50000 (Pos0) |
|  | | | | 30000 (Pos1) |
|  |  |  |  | 30000 (Pos2) |
|  |  |  |  | 23690 (Pos3) |
|  |  |  |  | 30000 (Pos4) |
|  | CHC | 50 pM (R4) | 15 mW | 10000 |
|  | PSD-95 | 100 pM (R6) | 15 mW | 10000 |
| Dataset_1_day2 | mEGFP-ARC | 60 pM (R3) | 15 mW | 30000 |
|  | CHC | 50 pM (R4) | 15 mW | 12500 |
|  | PSD-95 | 100 pM (R6) | 15 mW | 12500 |
| Dataset_2_CTRL_BrainPhys | mEGFP-ARC | 50 pM (R3) | 18 mW | 30000 |
|  | CHC | 50 pM (R4) | 18 mW | 10000 |
|  | PSD-95 | 200 pM (R2) | 18 mW | 12000 |
| Dataset_2_CTRL_Charu | mEGFP-ARC | 50 pM (R3) | 18 mW | 30000 |
|  | CHC | 30 pM (R4) | 18 mW | 15000 |
|  | PSD-95 | 150 pM (R2) | 15 mW | 15000 |

**Table S2. DNA-PAINT imaging parameters**

The table reports the parameters used during the imaging acquisition of DNA-PAINT experiments.

**Supplementary References**

1. Masullo, L.A., Boden, A., Pennacchietti, F., Coceano, G., Ratz, M., and Testa, I. (2018). Enhanced photon collection enables four dimensional fluorescence nanoscopy of living systems. Nat Commun *9*, 3281. 10.1038/s41467-018-05799-w.
